# Nondestructive material characterization and component identification in sheet metal processing with electromagnetic methods

**DOI:** 10.1038/s41598-024-55927-4

**Published:** 2024-03-15

**Authors:** Bernd Wolter, Benjamin Straß, Kevin Jacob, Markus Rauhut, Thomas Stephani, Matthias Riemer, Marko Friedemann

**Affiliations:** 1 Fraunhofer Institute for Nondestructive Testing IZFP, 66123 Saarbrücken, Germany; 2 Fraunhofer Institute for Industrial Mathematics ITWM, 67663 Kaiserslautern, Germany; 3 Fraunhofer Institute for Machine Tools and Forming Technology IWU, 09126 Chemnitz, Germany

**Keywords:** Energy science and technology, Materials science

## Abstract

Electromagnetic methods for non-destructive evaluation (NDE) are presented, with which sheet metal components can be identified and their material properties can be characterized. The latter is possible with 3MA, the Micromagnetic Multiparametric Microstructure and stress Analyser. This is a combination of several micromagnetic NDE methods that make it possible to analyse the microstructure in a ferromagnetic material and to determine quantitative values of the mechanical material properties or the stress state. In the case of cold forming, the 3MA application for pre-process testing of sheet metal is discussed. Based on the 3MA information, the formability of the sheets can be predicted. To apply 3MA in-line, the influence of the relative speed and the relative distance between the 3MA probe head and the sheet was investigated. In a second study, a spatially resolved eddy current (EC) method was used to create an image of the intrinsic material microstructure of a component for its identification and traceability. It turned out, that these intrinsic fingerprint images can still be recognized even after subsequent plastic deformation or coating of the surface. This enabled the development of a marker-free traceability method for sheet metal processing. It is based on a low-cost array sensor and a specimen identification using robust and partly redundant features of the fingerprint images processed by machine learning (ML).

## Introduction

Increasing production flexibility is one of the most important trends in the development of modern sheet metal forming^1^. The change from rigid production chains to flexible production steps and ever more challenging sustainability requirements increases the demand for new process monitoring and control strategies^2^. With regard to the material properties, open control loops have so far been used in sheet forming, i.e. there is no way to measure these properties in-line or even in-process and therefore there is no real-time feedback based on them^3^. However, flexible and agile manufacturing systems of the future require to comprehensively record critical material properties at each stage in production. Then, material data from the semi-finished products could be used as individual input variables for ongoing and downstream processes. Hence, material, energy and emissions could be saved through avoiding of waste and inefficiency.

In addition, future production also requires a comprehensive traceability concept^4^. Each production part with its individual characteristics and its actual conditions should be identifiable at any point within the entire production chain and beyond. This is an essential prerequisite for highly flexible and individualized production. Conventionally, for this purpose objects are marked with QR codes (Quick Response), laser markings, barcodes or RFIDs (Radio-Frequency Identification)^5^. The application of such artificial markers is an often time-consuming and costly additional process step and in most cases it is simply impossible for a single marker to remain as permanently readable information throughout all phases of the production process^6^. Ideally, natural and inherent characteristics of the component itself will be used for its identification. One possibility is to use the component's characteristic surface microstructure, which reflects its individual manufacturing history^7^. To do this, an optical camera is used to record a high-resolution image of a selected area of the component surface, from which a characteristic bit sequence is calculated and assigned to the component ID. In order to identify the component again later, the process is repeated and the captured image is compared with the database. It is clear that this approach only will work, if the majority of the surface remains visible and if it is changed only slightly during processing. However, if the component is subjected to process steps such as forming, machining or complete painting that significantly change the surface or even completely cover it, optical reading is no longer possible. Therefore, optical markers, whether artificial or natural, can only be used to a limited extent for traceability in sheet metal processing.

Electromagnetic testing includes a wide range of different non-destructive evaluation (NDE) methods. What they have in common is that they assess the condition of the test object by observing its electromagnetic reaction to electric currents and/or (electro-) magnetic fields generated in it^8^. It will be shown below that electromagnetic NDE offer solutions for both tasks described above, in-line material property assessment and traceability in sheet metal processing.

## Methods

All electromagnetic NDE methods are based on the set of four coupled partial differential equations described by Maxwell^9^. A detailed description of the physical basis of electromagnetic NDE was given by Nagy^10^. Eddy current testing (ET) and magnetic flux leakage testing (MT) are established NDE methods for detecting surface and subsurface defects^11,12^. However, it is also known that electromagnetic NDE, such as the so-called “micromagnetic” methods described below, can be used to characterize material properties as well as (applied or residual) stresses in the material^13^. These applications have intensively studied since many years, especially for ferromagnetic materials^14^.

### Electromagnetic method for sheet material characterization

**Figure 1:**
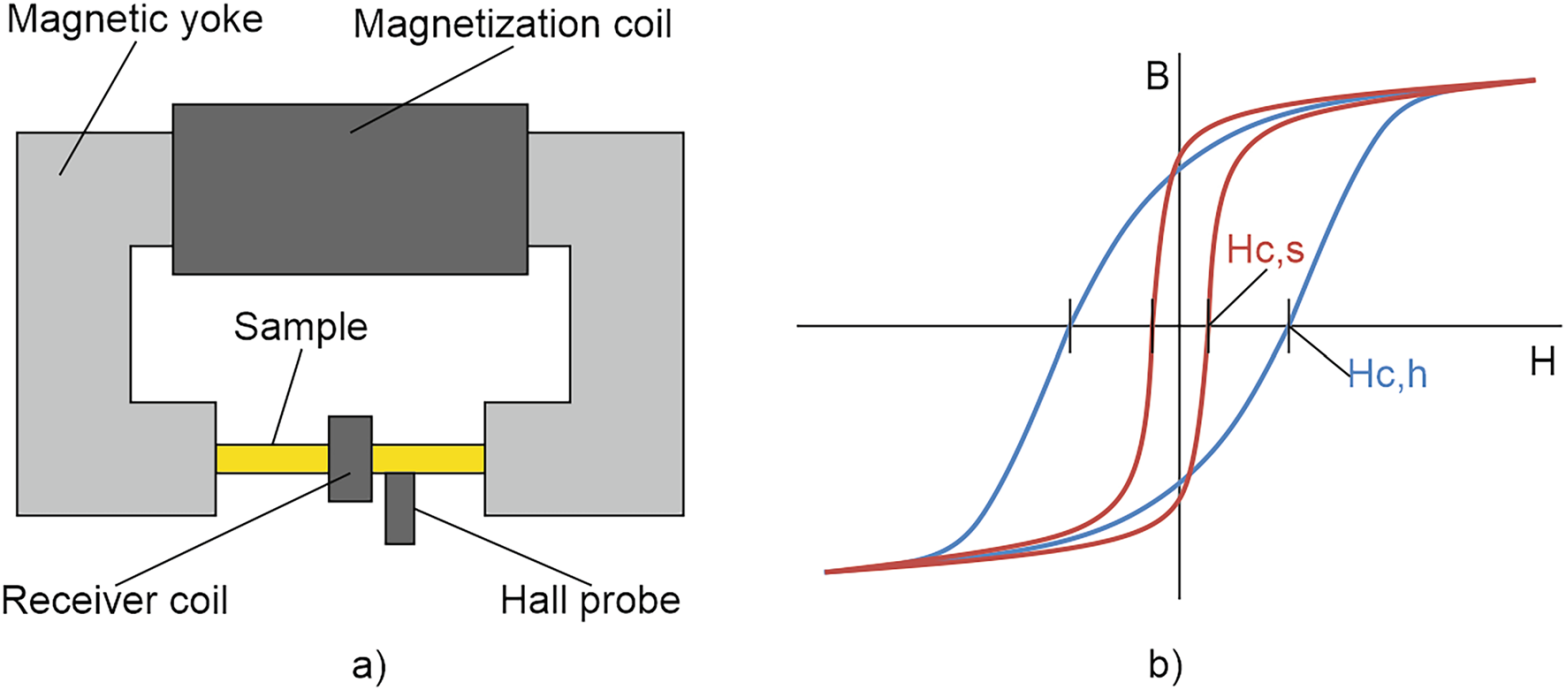
Magnetic hysteresis measurements; (**a**) Measuring set-up for a standard magnetic hysteresis measurement; (**b**) Magnetic hysteresis loops for a hard material (with coercivity *Hc,h*) and a soft material (with *Hc,s*).

Micromagnetic methods of electromagnetic NDE can be used to characterize material properties, as described in the result chapter “Sheet material characterization”. It’s been more than 80 years since the idea of using such methods to determine the mechanical properties of a material, which define its behaviour under the action of external mechanical load, was born^15^. With the exception of eddy-current (EC) measurement, the application of these methods is restricted to ferromagnetic materials. Such materials are characterized by magnetic domains separated from each other by so-called Bloch walls^16–18^. Now a limited volume of this ferromagnetic material is cyclic magnetized. That is, an alternating external magnetic field *H* is applied, which causes a rearrangement of the domain structure, which is accompanied by Bloch wall movements and, at higher fields, by the rotation of the magnetization of the domains towards the *H* field direction. Macroscopically, this periodic magnetization will result in a magnetic hysteresis curve, showing a non-linear relation between the magnetic flux density *B* in the material and the applied magnetic field strength *H* (see Fig. 1b).

Most ferromagnetic materials used in industry, such as steels, are polycrystalline materials with "lattice defects" in a broad sense, such as solute atoms, immovable dislocations, grain boundaries, and precipitations. In steel production such lattice defects are artificially created during work hardening, precipitation hardening, solid solution strengthening and grain refinement in order to improve the mechanical properties of the metal. This is because lattice defects hinder or even prevent the movement of dislocations during plastic deformation^19^. The same lattice defects also temporarily impede the movement of the magnetic Bloch walls^20^. Therefore, the magnetization process and the hysteresis behaviour of the material is influenced by its microstructure, which also affects the mechanical-technological properties of the material. Models that describe the relationship between microstructural properties of steels and their magnetic hysteresis are known from literature^21^. In addition, the magnetization process is also affected by the prevailing stress state in the material. Nevertheless, in many ferromagnetic materials (linear or non-linear) correlations can be observed between the mechanical-technological properties and the micromagnetic properties. Therefore, the later can be measured nondestructively in order to indirectly determine the former, if these correlations are known.

For example, harder steel grades typically have higher values of magnetic coercivity, *Hc* than softer steel grades (see Fig. 1b). In the magnetic hysteresis curve *Hc* can be determined as the value of *H* at the zero crossing of *B*. To determine the “true” *Hc* value, a material sample has to be placed a closed magnetic circuit, as shown in Fig. 1a. A homogenous magnetization of the sample is generated with a closed electromagnet, which consists of a magnetic yoke and an enclosing magnetization coil, operated at quasi-static conditions, i.e. at very low frequencies (e.g. *f* = 0.1 Hz). This ensures an uniform magnetic flux in the cross-section of the sample. A Hall probe is used to measure the component of the magnetic field *H* tangential to the sample surface, which corresponds to the value inside the sample. The magnetic flux density *B* is determined by using an enclosing receiver coil, in which a voltage signal proportional to the change in flux over time is induced.

**Figure 2:**
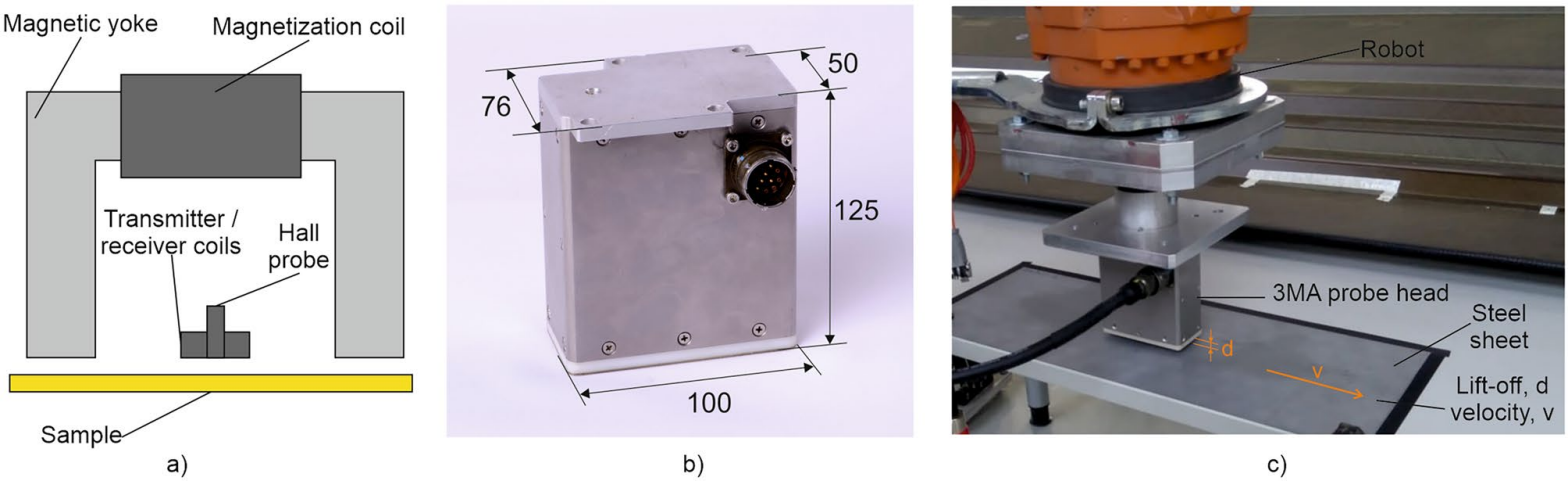
3MA technology for in-line sheet testing; (**a**) Principle construction of a 3MA probe head with an open U-shaped electromagnet; (**b**) Dimensioned image of the used 3MA probe head; (**c**) Measuring set-up for investigating the influence of variations of relative velocity v and lift-off d between probe-head and sample.

Standardized hysteresis measurements are not useful for in-line material characterization in metal sheet processing. They would require a sample extraction, i.e. the destruction of the test object. For in-line applications, a probe head concept with an open U-shaped electromagnet is required, such as that used in the 3MA technology (Micromagnetic Multiparametric Microstructure and stress Analyser). This probe head concept is shown in Fig. 2a. For the study described below, the 3MA probe head of Fig. 2b and the measuring set-up of Fig. 2c were used (see chapter “Results”). The 3MA technology combines four micromagnetic methods, namely multi-frequency eddy current (EC), harmonic analysis (HA) of tangential magnetic field, incremental permeability (IP) and magnetic Barkhausen noise (BN). These methods and the 3MA approach are described in detail elsewhere^22–24^.

The magnetic yoke of the 3MA probe head excites the low-frequency magnetization in the sample with a frequency f between 10 and 1,000 Hz and a high amplitude A up to 100 A/cm. A Hall probe and transmitter / receiver coils are attached in the middle between the pole shoes of the magnetic yoke. The coils are responsible for generating the high-frequency, low-amplitude magnetization for EC and IP as well as for receiving the signals from EC, IP and BN. The Hall probe is used to detect the tangential magnetic field (for HA) and to control the magnetization amplitude A. For an unambiguous determination of mechanical material properties, 3MA combines several measuring parameters from EC, HA, IP and BN (see Supplementary Information, Appendix 1). The parameters provide partly complementary and partly redundant physical information about the material being examined. By combining several of these parameters, quantitative values of a so called target quantity can be determined. A target quantity is a property or a feature of the material under investigation, which can be quantitatively determined with 3MA after it has been calibrated. Typical target quantities are hardness, hardening depth, characteristics of the tensile test (e.g. tensile strength, yield strength, fracture elongation, etc.), applied and residual stress or parameters of microstructure, like average grain size or content of martensite, retained austenite and cementite. Several target quantities can be determined at the same time. Prior calibration is required, which means that a model must be determined that describes the functional relationship between the target quantity and the 3MA parameters based on empirical data. To do this, the target quantity and the 3MA parameters must be measured on a set of calibration samples with graded values of the target quantity. The calibration model is then determined based on a regression analysis or other machine learning (ML) methods.

In the case of regression analysis, the calibration model can be written as

1 $${\text{y}}\text{ } = \, {\text{a}}_{0}\text{+} \, {\text{a}}_{1}\cdot {\text{x}}_{1}^{\text{q1}}+\dots +{\text{a}}_{\text{n}}\cdot {\text{x}}_{\text{n}}^{\text{qn}}$$

where ${\text{y}}$ is the target quantity ${\text{x}}_{\text{i}}$ are the 3MA measuring parameters and ${\text{qi}}$ their modifiers. Usually the ${\text{qi}}$ are limited to the values 1, 2 or 1/2. The coefficients ${\text{a}}_{\text{i}}$ (*i* = 1,…, *n*) have to be determined by a least squares error minimization and *n* stands for the dimensionality, i.e. the total number of 3MA parameters (incl. modifiers) used in the model.

### Electromagnetic method for sheet metal part identification

The following describes the electromagnetic method used for the results in the chapter “sheet metal part identification”. In sheet metal processing a reliable method is needed to identify an individual metallic component before forming and recognize it again after forming, respectively after surface coating. In principle, spatially resolved measurements with the micromagnetic methods described above could be a possible solution for this task. Such measurements are suited for imaging the irregularities of the material microstructure beneath the surface up to a skin depth between a few tenths of a millimetre and a few millimetres, depending on the magnetization frequency *f* and the electromagnetic material properties, i.e.  the electric conductivity σ and the magnetic permeability *µ*_*r*_. These of the intrinsic microstructure features can be used as an unique “fingerprint image” in order to identify the component.

A similar approach was already described by D. Mascareñas et al.^25^. They scanned ferromagnetic components with a Barkhausen noise (BN) probe head, which consists of a U-shaped electromagnet for magnetization and a pick-up coil for the BN signals. However, this approach has limited applicability for sheet metal processing for several reasons. Since the BN signal is very sensitive to lift-off fluctuations, the probe head was pressed to the sample using a pressure spring throughout the entire measurement. It is obvious that this is difficult to achieve with industrial sheet metal or already formed components.

**Figure 3:**
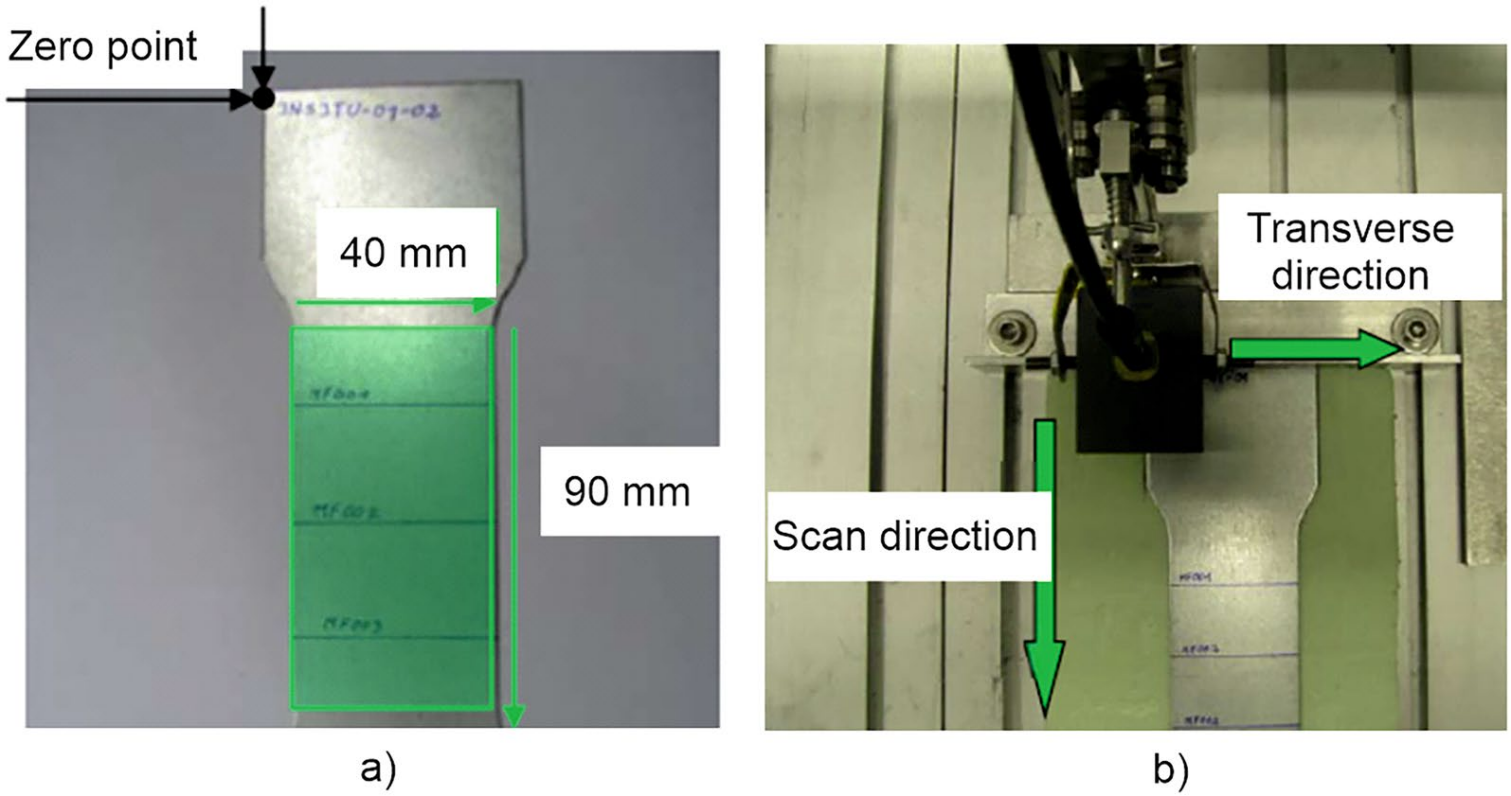
Eddy current measurements for component identification; (**a**) modified tensile samples and (**b**) measuring set-up with scanning robot.

Own investigations began with scanning 3MA measurements on modified tensile specimens cut from sheets (see Fig. 3a). Results are shown in appendix 3 of the Supplementary Information. However, in later studies the eddy-current (EC) method was exclusively used to generate fingerprint images. This is justified as follows. EC does not require an electromagnet, thus allowing simple construction of probe head arrays. EC is generally also applicable on electric conductive materials that are not ferromagnetic, i.e.  basically also on aluminium sheets. Additionally, because low-frequency yoke magnetization is not required, EC measurements are faster than the other micromagnetic methods. Therefore, 2D meander scans were subsequently performed using a conventional single-coil EC system, as shown in Fig. 3b.

**Figure 4:**
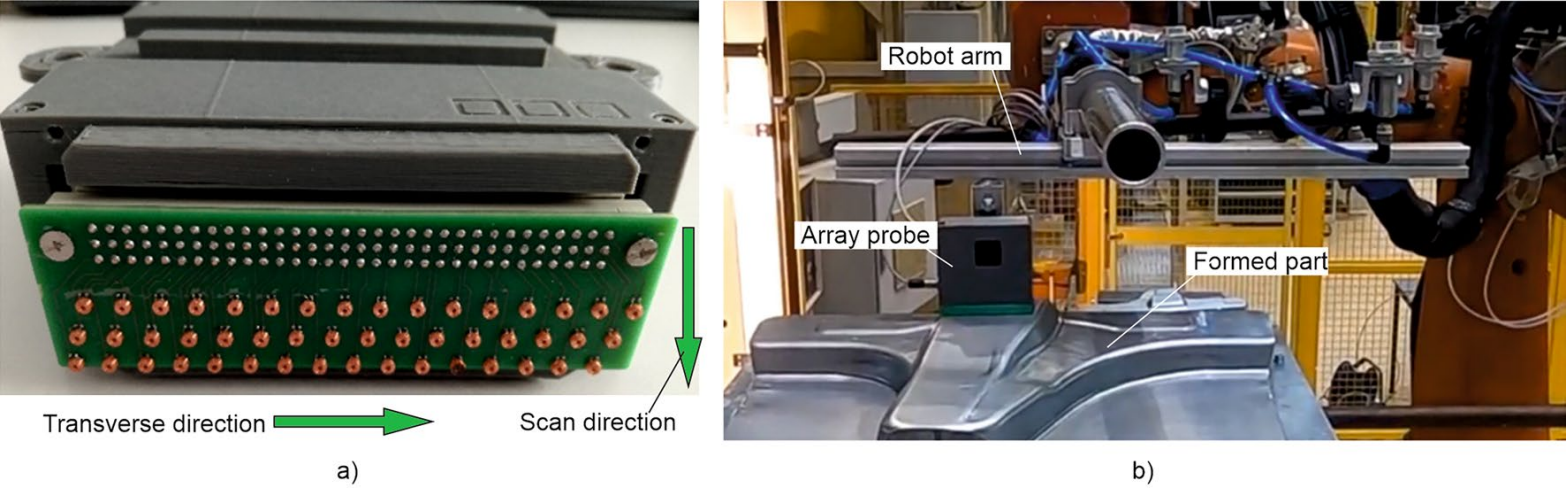
Equipment for fast measurement of electromagnetic fingerprint images; a) Multi-coil array probe; b) Robot guided measurement with the array probe at a B-pillar base.

The meandering 2D scans with a single-coil EC sensor are very time-consuming. A single fingerprint image takes several minutes and therefore this approach is not suitable for industrial production. For that reason, a new array probe based on an customized 48-fold sensor array was developed, which is shown in Fig. 4a. Each coil has an outer diameter of 2.5 mm. The coils are arranged in three rows of 16 coils on an area of 20 × 100 mm^2^. The distance between coils is 6 mm. From line to line the coils are offset from each other by 2 mm, resulting in a pixel resolution of 2 mm in the scanning direction. In addition, the classic EC device technology has been replaced by a cost-effective alternative, namely an LDC (Inductance to Digital Converter) from Texas Instruments®. In case of an LDC, the electronic components required to measure the energy dissipation and resonant frequency of an LC resonator are integrated on a microchip^26^. The array probe was attached on a robot arm for use in an industrial setting (see Fig. 4b). This set-up was used for measurements on blank sheets and on formed parts, e.g. on B-pillar, which were automatically scanned before and after forming.

**Figure 5:**
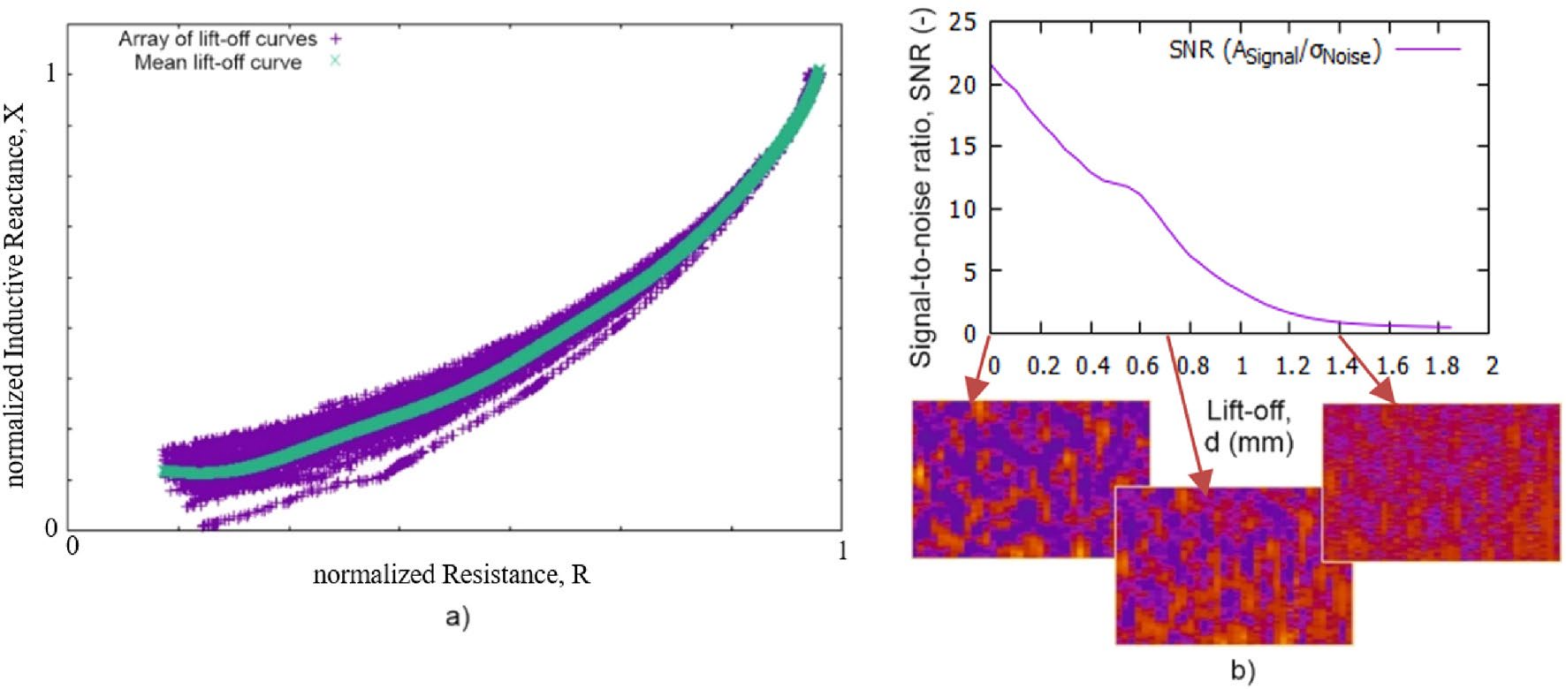
Lift-off compensation; (**a**) Determination of the mean lift-off curve for lift-off compensation in the array probe, (**b**) signal-to noise ratio (*SNR*) as a function of lift-off *d* and associated fingerprint images of a sample at *d* = 0.0, 0.7 and 1.4 mm. The *SNR* here is defined as the ratio of the mean absolute signal amplitude to the standard deviation of the noise. The scale is linear.

Due to the rigid structure of the array probe, local unevenness in the sample cannot be compensated. A variation of the lift-off from coil to coil within the array and thus local signal fluctuations could be the result. Therefore, a method was developed that aims to compensate for the measurement effects due to lift-off variations. First, the measuring system must be trained on a single reference sample from the sheet material being examined. For this purpose, numerous measurements are carried out at different positions of the reference sample by measuring the position-dependent lift-off signals. This means that for each coil the change in the complex-valued impedance ${\text{Z}} \, = \text{ } {\text{R}}\text{ + }{\text{iX}}$ is recorded, as the array probe is gradually lifted from the material. This data is then shifted to a quadrant of the coordinate system and converted to polar coordinates $\left( {{\text{R,}}\,{\text{X}}} \right)\, \to \,\left( {\overline{Z}{,}\,\phi } \right)$, where $\stackrel{\mathrm{-}}{\text{Z}}$ is the absolute value of ${\text{Z}}$ and $\phi$ is its phase angle. Now $\widehat{\phi }\text{(}\stackrel{\mathrm{-}}{\text{Z}}\text{)}$, the mean lift-off curve, is determined via regression as a function of the absolute value $\stackrel{\mathrm{-}}{\text{Z}}$ as shown in Fig. 5a.

After this training, $\widehat{\phi }(\stackrel{\mathrm{-}}{\text{Z}})$ is used to calculate the lift-off corrected signal images as follows. Given a spatially resolved grid measurement ${({\text{R}}, {\text{X}})}_{\text{k,l}}$, where $k,l$ are the pixel indices for each complex valued pixel ${({\text{R}}, {\text{X}})}_{\text{k,l}}$. Again a transformation into polar coordinates takes place, ${({\text{R}}, {\text{X}})}_{\text{k,l}} \, \to \, {(\stackrel{\mathrm{-}}{\text{Z}}, \phi )}_{\text{k,l}}$ Then the difference between the measured angle ${\phi }_{\text{k,l}}$ and the angle of the mean lift-off curve $\widehat{\phi }({\stackrel{\mathrm{-}}{\text{Z}}}_{\text{k,l}})$ is calculated, which corresponds to the lift-off corrected pixel value ${\text{u}}_{\text{k,l}}$ measured at this position, i.e.

2 $${\text{u}}_{\text{k,l}}\text{ = }{\phi }_{\text{k,l}}\text{ - }\widehat{\phi }({\stackrel{\mathrm{-}}{\text{Z}}}_{\text{k,l}})$$

With this approach it is possible to compensate for lift-off fluctuations up to *d* = 1 mm. Above 1 mm, the signal-to-noise ratio (*SNR*) is too low (< 4) for compensation, as shown in Fig. 5b. The *SNR* is calculated as the ratio of the mean absolute signal amplitude to the standard deviation of the noise. For *SNR* < 4 the fingerprint images are too low-contrast for further processing. At this point, temperature-related signal fluctuations were not taken into account. Since the calibration function was determined at a fixed temperature, it is not fully applicable at other temperatures.

The lift-off compensated fingerprint image, which represents the component as an unique individual, is stored in a feature space. In order to unambiguously identify these fingerprint images, special image processing methods are applied now. Different approaches were tested. First, a direct comparison of the images by using an image correlation technique was applied. Since plastic deformation can occur, this correlation analysis was combined with an optical flow estimation^27^. The classifier used was based on a combination of optical flow to account for the deformation and the Euclidian distance for the actual comparison. The optical flow between the two pictures, i.e. ${u}^{(1)}={u}^{\left(1\right)}\left(k,l\right)={u}_{k,l}^{(1)}$, and ${u}^{(2)}={u}^{\left(2\right)}\left(k,l\right)= {u}_{k,l}^{(2)}$, was determined with Farnebäck’s algorithm^28^. Again $k,l$ are the pixel indices. From this, the Euclidean distance of the reconstruction of the second measurement (first measurement + optical flow) was calculated, which should be small when comparing two measurements of the same specimen and large when comparing measurements from different specimens are compared. The Euclidian distance between the first image and the reconstruction is given as

3 $${\Vert {u}^{(1)}-{u}^{(Reconstruction)}\Vert }_{2}=\sqrt{\sum_{k, l}{\left({u}^{\left(1\right)}\left(k,l\right)-{u}^{\left(Reconstruction\right)}\left(k,l\right)\right)}^{2}},$$

where ${u}^{(Reconstruction)}\left(k,l\right)={u}^{\left(1\right)}\left(k-{v}_{k}\left(k,l\right),l-{v}_{l}\left(k,l\right)\right)$ and $v$ is the optical flow displacement field. If ${u}^{(1)}$ and ${u}^{(2)}$ represent fingerprint images of the same specimen before and after forming, the optical flow displacement field corresponds to the plastic deformation that the specimen underwent and can be used to reconstruct the second image from the first (${u}^{(2)}\approx {u}^{(Reconstruction)}$). Therefore, for measurements on identical specimen before and after plastic elongation, the Euclidian distance should be small. When comparing images from different specimen, the second image can only be inadequately reconstructed from the first using the displacement field, which should lead to a large Euclidean distance. This assumption is confirmed in the results.

On the other side, this “correlation analysis with optical flow” algorithm is only suitable for small data sets and simple plastic deformation. Therefore, another approach was to use low-level scale-invariant features, e.g. so-called SURF features in combination with machine learning (ML) methods, such as random forest classifiers^29^. The advantage over correlation analysis is that the image is not compared pixel by pixel, but based on characteristic features. In recent years, Machine Learning (ML) has established itself as a successful tool for image analysis and computer vision, especially for classification tasks^30,31^ and object recognition^32,33^. Classification results based on these features have shown promising results, but tend to be unstable and do not translate well to new images.

Ultimately, Convolutional Neural Networks (CNN) were used, in which not only the classifier but also the features are trained based on data. This approach has also provided better and more stable results in other image classification tasks^34^. Now, these deep neural networks have proven to be successful in many kinds of image processing applications^35,36^. Traditional CNNs are used to train a classifier. This means that the desired output classes must be known in advance. In contrast, Siamese Convolutional Networks (SCNNs) represent a special form of a neural network architecture in which a similarity function between images is trained. In the application shown here there are no fixed classes, but rather new instances each time, which is why a Siamese network is advantageous here instead of a standard CNN. SCNNs have already been used for similar purposes^37,38^.

**Figure 6:**
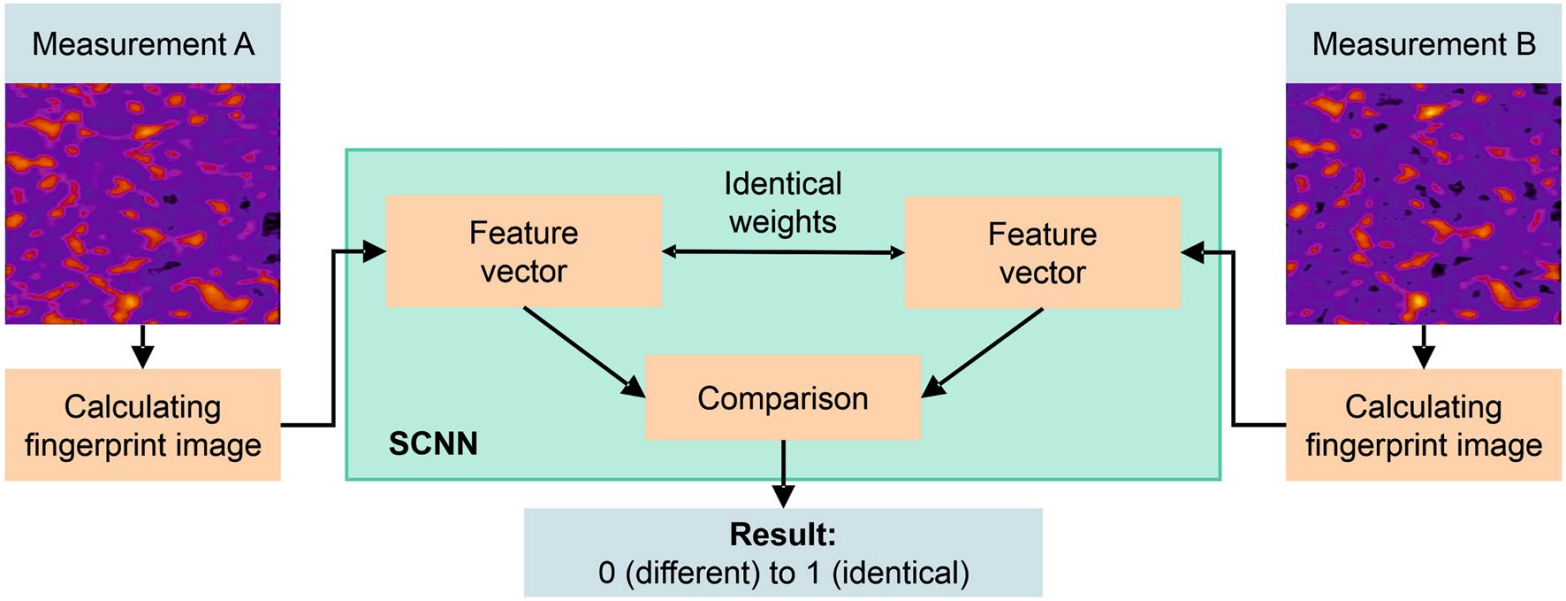
Functional principle of a Siamese Convolutional Neural Network (SCNN). Two measurements are compared, either from different specimens or from the same specimen and after different production steps. The output is a number between “0” (measurement A and measurement B come from different specimens) and “1” (measurement A and B come from the same specimen).

The basic functionality of the used SCNN is shown in Fig. 6. To determine whether two measurements ("Measurement A" and "Measurement B") come from the same specimen, the lift-off-compensated fingerprints (${\text{u}}_{\text{k,l}}$) are first calculated as described above. These fingerprints now represent the input of the SCNN. The SCNN itself consists of three subnetworks, the first two being identical and responsible for extracting a feature vector from each of the two fingerprints, which are then compared by the third subnetwork, which acts as a regression network. The output is a number between 0 and 1, where 0 represents no match (the two fingerprints come from different specimens), and 1 represents a match (the two fingerprints come from the same specimen). The transition to SCNN can also be justified by its ability to learn the typical changes resulting from plastic deformation from the provided training data, instead of estimating them as in the case of the optical flow based method.

One of the biggest challenges in applying deep learning methods to real-world applications is the large amount of data required. The procedure for acquiring this amount of data is described below. On 16 plane sheets (which later were formed to rear light pots), 3 measuring areas were determined in advance in each case. 120 repeated measurements were carried out on each of these measuring areas, varying various parameters. The parameter lift-off *d* was varied between 0 and 1 mm. The feed rate at which the measurement areas were scanned was varied from 50 to 500 mm/s. In addition, the angle (within a plane parallel to the measuring area) was varied from − 20° to + 20°. By this 5,760 fingerprint images were measured in total. In order to provide a sufficient amount of data as well as a sufficient amount of variance within the data, so-called data augmentation was applied, i.e. additional training data were created synthetically based on existing training data. In addition to photometric augmentations (brightness, contrast, gamma, sharpness, noise) and geometric augmentations (perspective transformations, non-linear distortions), problem-specific transformations (stretching, compression) were also used^39^.

To store and structure the recorded data (set-up parameters, raw signals, fingerprint images and extracted features), a digital object memory based on Semantic Web technologies was implemented, which represents the data as a link of self-descriptive elements. In order to enable identification, even if only some of the characteristic features of the electromagnetic images can be detected, stable and partially redundant features are generally used to enable identification. Identification is supported by a digital object memory and the additional use of process and quality data of the specimen added to the feature data space. Ultimately, the fingerprint sensor system includes a cloud-based data management system for managing and tracing the fingerprint data.

## Results

### Sheet material characterization

The aim of the following work was to use the 3MA technique in order to predict the forming behaviour of a steel sheet for a feed-forward control of its forming process. For this purpose, several mechanical material properties should be known. Hardness, yield strength, tensile strength and percent elongation are required to assess sheet metal formability. For most steels the *r*-value and the* n*-value should also be known.

The *r*-value, also called the plastic strain ratio, describes the tendency of the material to flow out of the sheet plane or out of the sheet thickness.The *n*-value is the work hardening exponent and is mathematically defined as the slope of the true stress—true strain curve in a double logarithmic coordinate system. 3MA was calibrated to all these target quantities.

For calibration, 25 steel sheets of 1 mm thickness and 700 × 500 mm^2^ area were used. The steel material was a galvannealed dual phase steel. The reference values of the target quantities were determined using destructive tests. An off-line 3MA calibration was used, i.e. the 3MA measurements for calibration were carried out under stationary conditions, i.e. without relative movement between steel sheet and 3MA probe head (*v* = 0 m/s). The lift-off (distance between probe head and sheet) during calibration measurement was *d* = 5 mm. The magnetization frequency and amplitude of the 3MA measurements were *f* = 200 Hz and *A* = 30 A/cm (for all methods). The calibration functions had a dimensionality of *n* = 9. The calibration functions and the parameters included therein are described in Appendix 2 of the Supplementary Information. The statistical characteristics of these calibration functions are presented in Table 1.

**Table 1:** Results of 3MA calibration to different mechanical material parameters.

Material parameter	Correlation coefficient, *R*^*2*^	Root-mean-square error, Δ*y*
Hardness, *H*	0.975	1.9 HV
Yield strength, *Rp0.2*	0.996	4.4 MPa
Tensile strength, *Rm*	0.995	1.9 MPa
Elongation, A	0.974	0.7%
r-value	0.983	0.032
n-value	0.987	0.003

Each row in this table shows the correlation coefficient *R*^*2*^ and the root-mean-square error Δ*y* of the correlation between calibrated 3MA values and destructively tested reference values. The off-line calibrated 3MA system was then applied in-line to moving sheets. The influence of the relative velocity *v* and variations of lift-off *d* on the measurements with the calibrated 3MA system was now investigated, as both parameters can vary during in-line application. For this purpose, the measuring set-up in Fig. 2c was used. A strong robot moves the probe head over the sheet with different lift-offs *d* and different velocities *v*. The reference point was always the initial “calibration situation” with *v* = 0 m/s and *d* = 5 mm.

**Figure 7:**
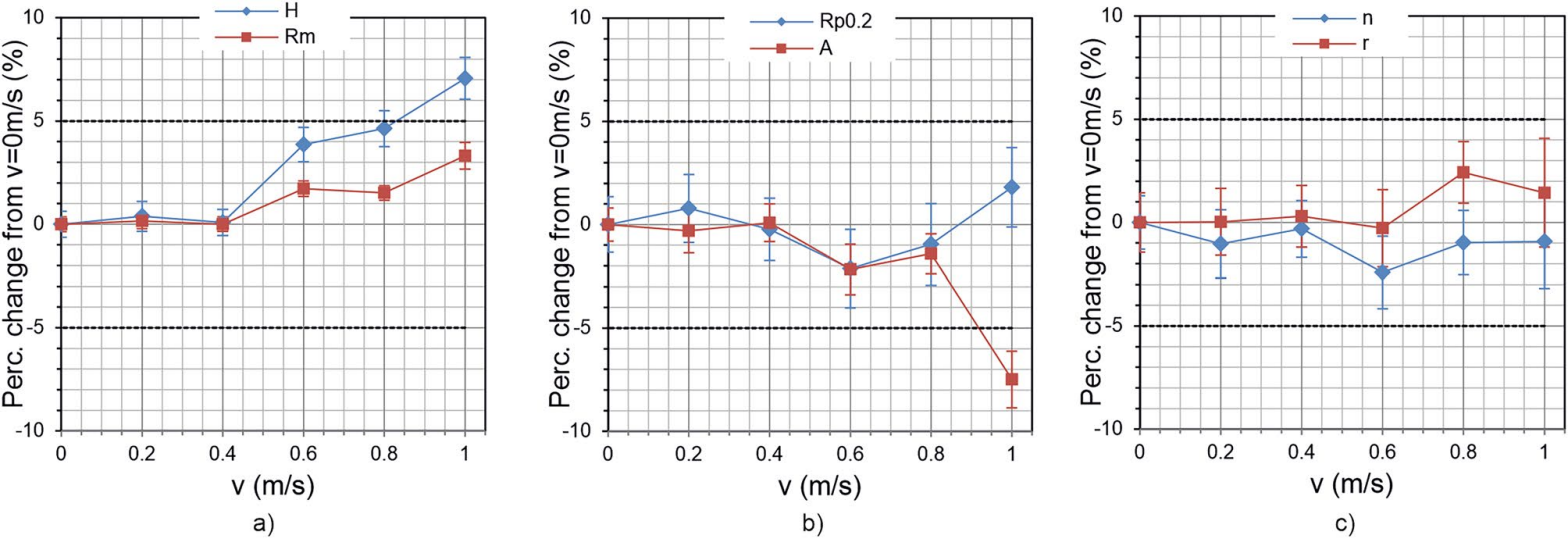
Influence of velocity variation; Percentage change from the value measured at *v* = 0 m/s as a function of feed velocity *v* for (**a**) hardness *H* and tensile strength *Rm*; (**b**) yield strength *Rp0.2* and percentage elongation *A*; (**c**) *r*- and *n*-value.

**Figure 8:**
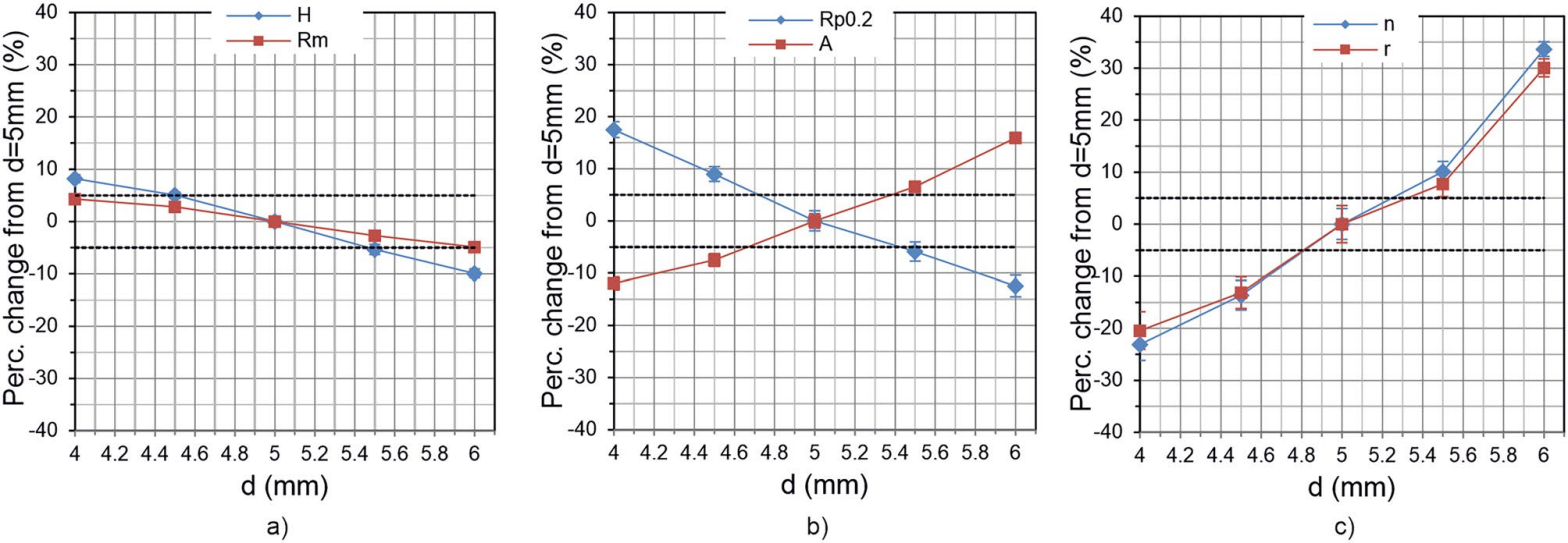
Influence of lift-off variation; Percentage change from the value measured at *d* = 5 mm as a function of lift-off *d* for (**a**) hardness *H* and tensile strength *Rm*; (**b**) yield strength *Rp0.2* and percentage elongation *A*; (**c**) *r*- and *n*-value.

Figure 7 shows the percentage change of the target quantities values, when *v* is varied between 0 and 1 m/s, which is a typical range for the sheet transport velocity in industrial environments. For comparison, the error bars at the measuring points show the percentage deviation within a measurement at constant *v*. The dashed lines in the diagrams show the + / − 5% variation limit, which is accepted as the usual permissible margin of error in sheet steel processing. The same procedure was used to lift-off *d*. These results are shown in Fig. 8.

### Sheet metal part identification

This section describes the results of the (marker free) part identification. Initial measurements were carried out on modified tensile specimens cut from sheets. The sheets were made of steel grade HC280LAD with a thickness of 1.2 mm. The measurement setup consisted of a robot that guided a 3MA probe head in a 2D meandering scan over an area of x*y = 40 mm*90 mm on the tensile samples. The dot spacing was 0.1 mm in the transverse direction and 0.25 mm in the scan direction. A total of 50 samples in their initial states (undeformed) have been used for teaching the equipment. Based on these measurements the feature data space was created. Subsequently, 10 of the samples have been stretched up to different degrees of permanent elongation. i.e. 2.5%, 5.0%, 7.5% and 10.0% elongation. The plastically stretched samples were measured again to see if they can still be recognized based on their electromagnetic signals. Initially, parameters from all four  3MA micromagnetic methods were used for these investigations. Results from all 3MA parameters are shown in Appendix 3 of the Supplementary Information. The first evaluation showed that the highest recognition rate after plastic deformation can been achieved with the measuring parameters from the EC method. The parameters of the other micromagnetic methods were less suitable, probably due to their higher sensitivity to residual stress, which is considerably changing during plastic deformation^40^.

**Figure 9:**
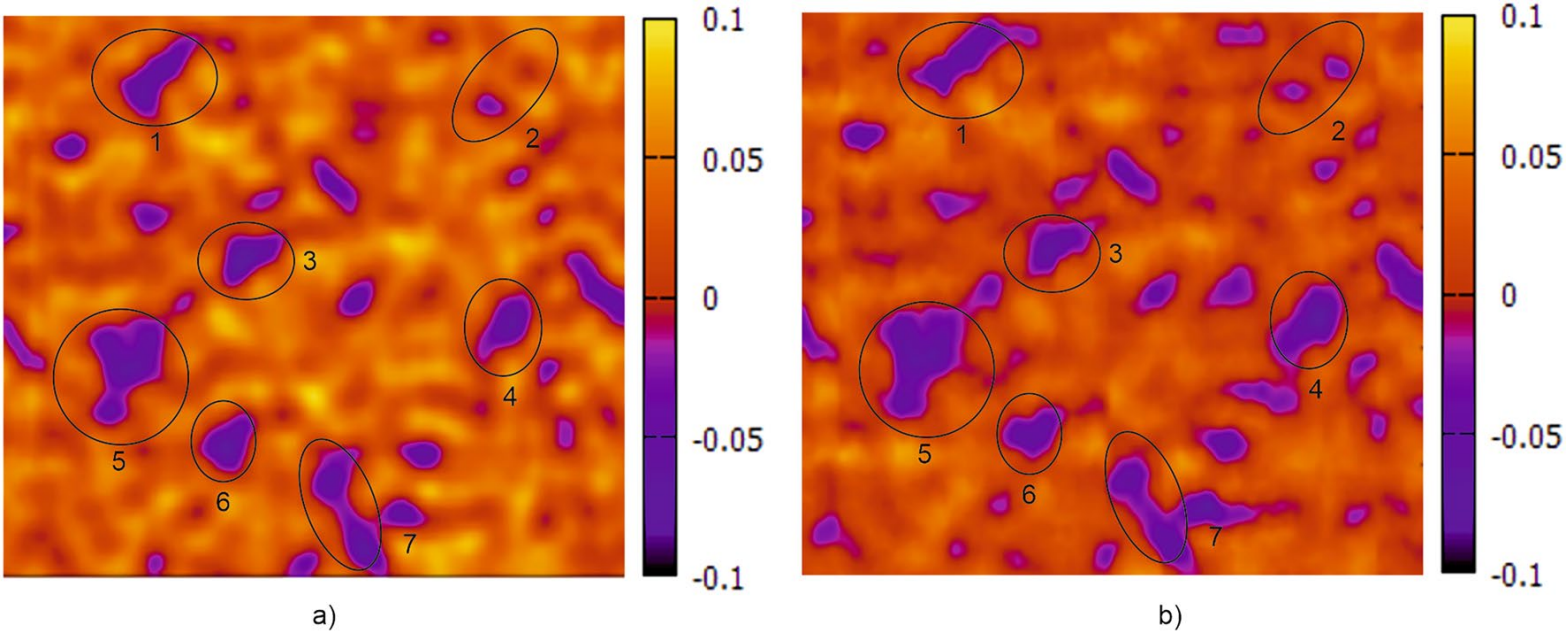
Electromagnetic fingerprint images: (**a**) result of a 2D scan a conventional EC; (**b**) result of a 2D scan with the low-cost LDC probe of the same area. In both cases the scanned area is approximately x*y = 50 mm*40 mm.

As already explained in chapter “Methods”, there were also other, more practical reasons to use EC only for further investigations. The pictures in Fig. 9 show some of the first EC fingerprint images. Both images show results from the same sample. Figure 9a shows the fingerprint image captured from a conventional EC 2D scan with a single coil. Figure 9b shows the result of a fast 2D scan with the low-cost array probe.

**Figure 10:**
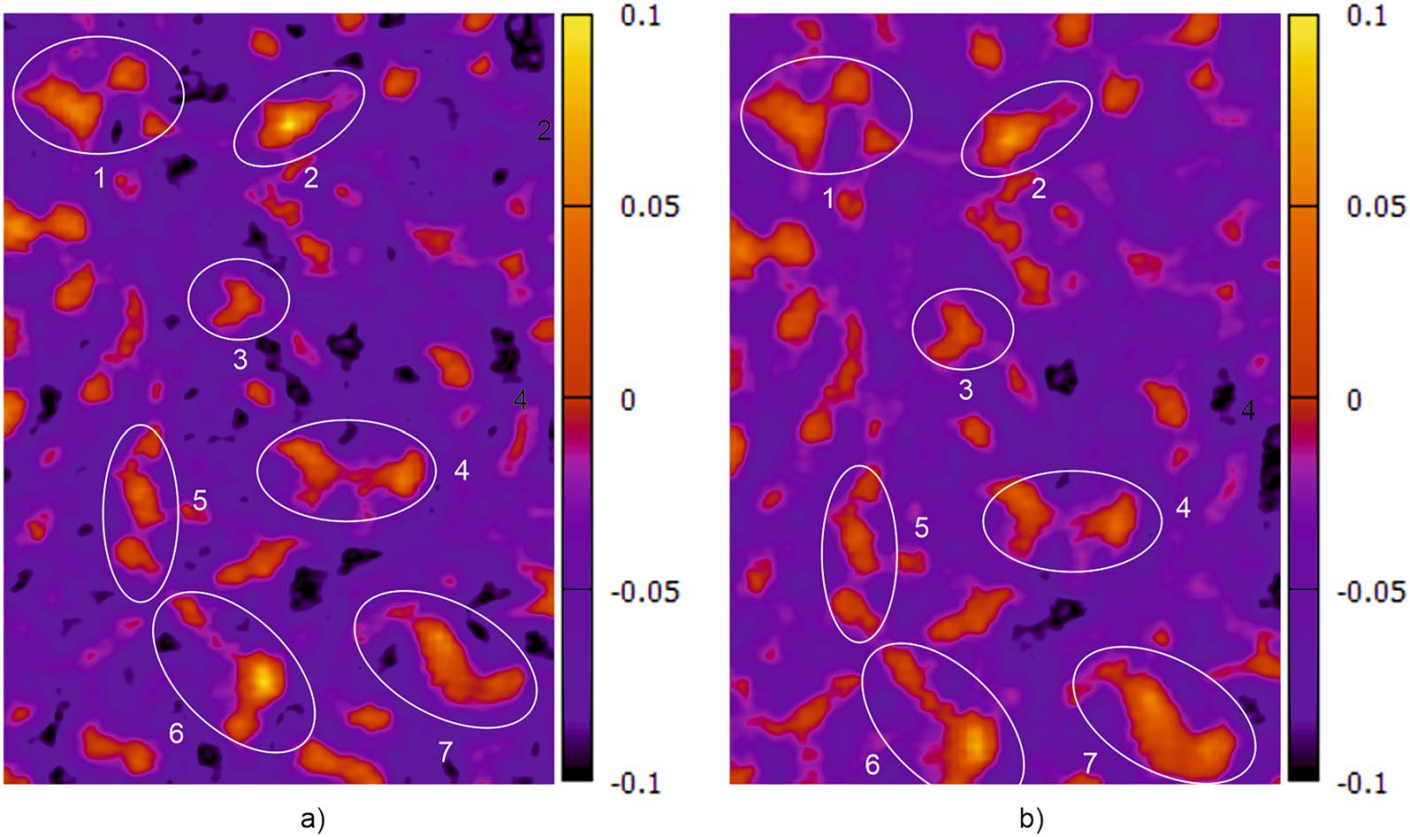
Electromagnetic fingerprint images of a stretched sheet sample: (**a**) before and (**b**) after 7.5% plastic elongation. The scanned area is approximately x*y = 50 mm*80 mm.

Some “fingerprint features” visible to the human eye in the images are encircled and numbered. However, this does not mean that exact these encircled features will be used by the machine learning (ML) algorithm to determine the feature vector. However, it demonstrates the similarity of the measuring information of both images. The pictures in Fig. 10 show the electromagnetic fingerprint images of a tensile sample before (a) and after (b) 7.5% plastic elongation.

Here too, some “fingerprint features” are encircled and numbered. What is interesting is that the visible features have been moved and their shape has been slightly changed. However, they are still detectable for the naked eye and also for the ML algorithm. Based on the optical flow method described in chapter “Methods” (see Eq. (3)) the specimen measured before stretching have been identified after stretching.

**Figure 11:**
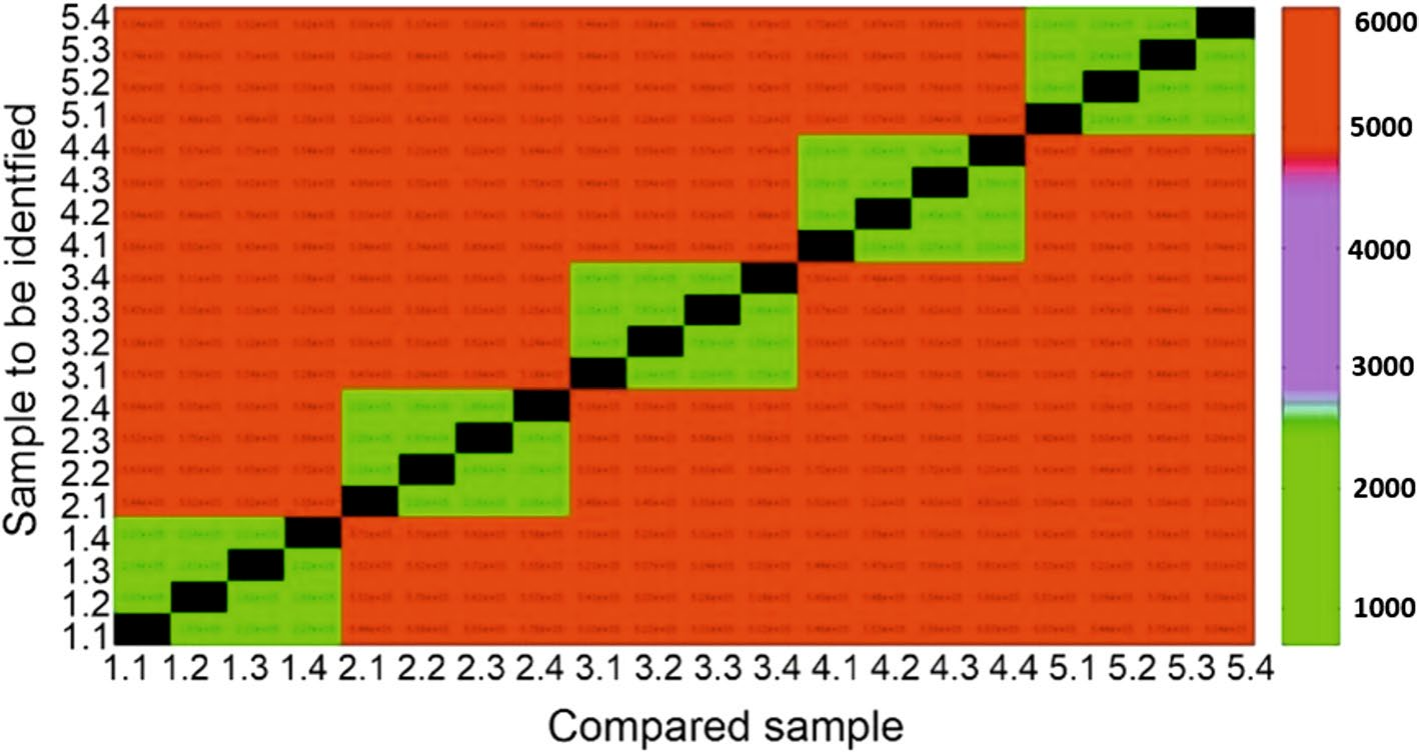
Colour-coded Euclidian distances for the comparisons: 1.1, 1.2, … show repeated measurements on the same sample, 1.1, 2.1, … show measurements on different samples.

Figure 11 shows the colour-coded Euclidian distances between different samples and identical sample, before and after stretching. The Euclidian distances for 5 samples and 4 repeated measurements per sample are shown as examples. Identifiers of type *n.m* are used in order to describe the measurement at sample number *n* and at repetition number *m*. These notations are used for the *x*- and *y*-axis. Green colour represents low distances and red colour large distances, while black colour indicates that the measurements were compared with themselves (“Euclidean distance” = 0). It can be observed, that all samples were identified correctly and in addition there is a large gap between the distances of repeated measurements on the same sample to the distances of measurements on different samples.

**Figure 12:**
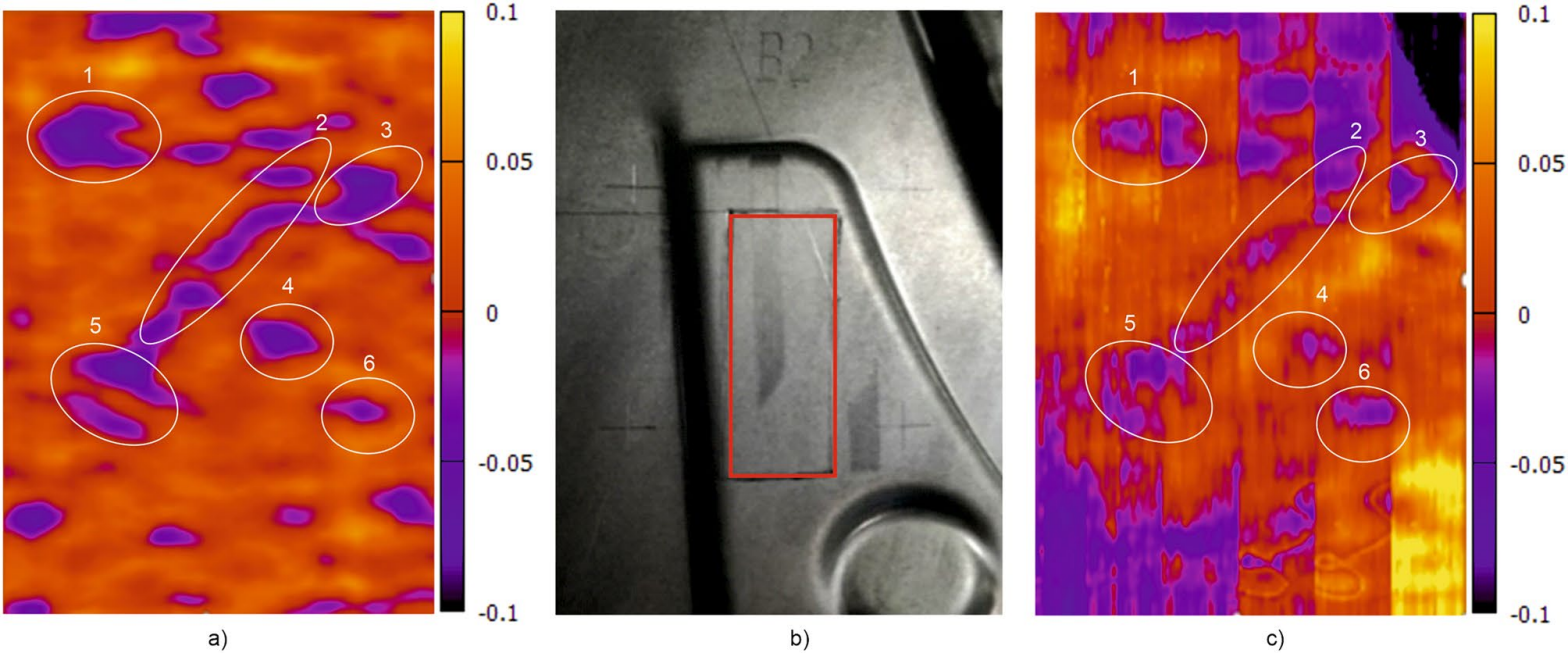
Application on industrial formed parts; (**a**) Electromagnetic fingerprint image (${\text{u}}_{\text{k,l}}$ from Eq. (2), the colour represents the value of ${\text{u}}_{\text{k,l}}$ at that pixel) from blank sheet; (**b**) Formed component with marked scan area; (**c**) Electromagnetic fingerprint image from the formed component.

**Figure 13:**
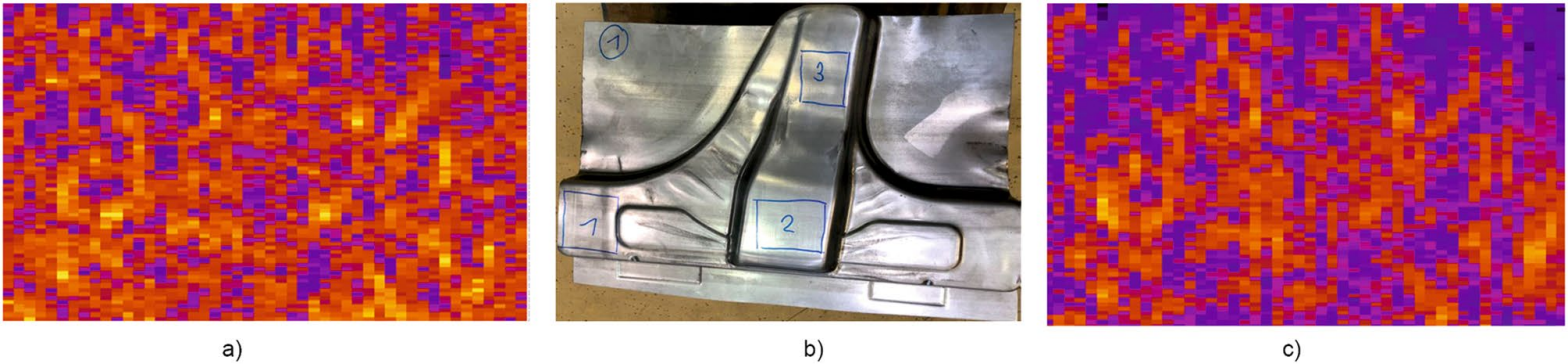
Application on industrial formed parts; (**a**) Electromagnetic fingerprint image from blank sheet; (**b**) Formed component with marked scan area no. 2; (**c**) Electromagnetic fingerprint image from the formed component.

As described above, a Siamese network (SCNN) was used for larger data sets. In this study test measurements were carried out on formed parts from industrial production. First, 16 flat blanks (before forming) were measured, each at 3 selected scan areas. In these scan areas the local plasticity was homogeneous, so that the resulting fingerprint images remained as similar as possible despite deformation of the component. Each blank was scanned 120 times,  varying the lift-off (0 to 1 mm), feed speed (50 to 500 mm/s) and scanning angle (− 20° to + 20°). The recorded data were stored in the digital memory with the parameters used in each case. Some measurement results are shown in Figs. 12 and 13.

In both cases the left images (a) show the electromagnetic fingerprint image captured in the selected scanning area of a blank. The blanks were then formed into pressed components and scanned again on the previously selected scanning areas. The middle pictures (b) show the formed component with scanning area (red rectangle in Fig. 12b and rectangle no. 2 in Fig. 13b). The right images (c) show the electromagnetic fingerprint images of the formed components made from the corresponding blanks. Only in case of Fig. 12 it is also possible to visually recognize some similarities between the fingerprints of the blank (a) and formed component (c). Nevertheless, most of the formed components could be traced back to the blanks by using SCNN, but the recognition rate was below 90%. It was observed that this poor recognition was due to a temperature-related signal drift, which was not yet compensated for at this time. With the sensor technology used, it could also be possible to display the local strain of the material in a spatially resolved manner, but this is not investigated here.

Finally, the application on painted sheets was investigated. Again, sheets, that have been imaged in their initial states should be still recognizable after processing, in this case after complete coating of the surface. In such a case it is obvious, that optical methods are completely unsuitable. But on the other hand, here the recognition rate of the electromagnetic method was excellent again, since the coating behaves just like a lift-off in the measurement signals. The prediction score after painting was above 99.9% for all parts.

## Discussion

The first part of the results showed how the 3MA system can be used for material characterization of sheet steel. All mechanical properties of the material relevant to assessing formability could be determined by using 3MA measurements (see Table 1). The observed linear correlations between predicted and destructively tested mechanical properties were excellent. Depending on the material parameters, correlation coefficients *R*^*2*^ between 0.974 and 0.996 have been observed. As already found in other studies, the worst correlation was determined for elongation *A*, which is probably due to the high relative error in the reference measurements.

An important result was observed that it is possible to perform an off-line calibration under stationary and constant conditions (*v* = 0 m/s, *d* = const.) applicable for later in-line measurements (*v* > 0 m/s, *d* ≠ const.), if the variations of *v* and *d* are moderate. This makes the application of 3MA much easier, as the measurements for calibration can be done in the laboratory. In the considered velocity range between 0 and 1 m/s, the permissible deviation limit of +/− 5% is only slightly exceeded at *v* = 1 m/s (see Fig. 7). However, it should be noted that the maximum permissible velocity is additionally limited by the sheet length *l*_*S*_ and the time for a 3MA measurement *t*_*3MA*_, i.e. ${\text{v}} \, \le \, {\text{l}}_{\text{S}}/{\text{t}}_{\text{3MA}}$ must also be fulfilled. In this study *t*_*3MA*_ was approximately 0.7 s and *l*_*S*_ was 700 mm, limiting *v* to 1 m/s. On the other hand, even small variations in the lift-off *d* seem to have a strong effect on the measuring results, especially for *r*- and *n*-values. In order to keep the values within the +/− 5% limit, the *d* variation should not exceed +/− 0.2 mm. For 3MA in-line applications, this can be achieved by stabilizing the sheet vibration during the measurement by using guiding rollers.

In the second part, eddy-current (EC) method was used for sheet metal part identification. In Fig. 9 it was demonstrated, that electromagnetic fingerprint images can be captured either by conventional EC equipment or by a low-cost alternative with a fast measuring sensor array. The images are almost identical and show characteristic features that are unique to each sheet component and therefore suitable for its identification. These features origin form intrinsic microstructure heterogeneities and they are still detectable after stretching, i.e. plastic deformation (see Fig. 10). However, the exact physical origin of these variations is not yet clear. It is obvious that they reflect microstructure features affecting the EC impedance, like local variations of electrical conductivity σ and magnetic permeability *µ*_*r*_. Other “irregularities”, like small cracks, pores and inclusions could also contribute to the contrast of the fingerprint images. On the other hand, the optically accessible surface microstructure does not seem to have a significant effect on the images. This was confirmed by comparing the electromagnetic images with optical images of some samples and no correlation could be found. This and the fact that the features are still detectable after painting / coating confirm that they originate from sub-surface material features.

One way to check whether a fingerprint image comes from the same component (e.g. before and after stretching) or not is to use an algorithm based on a correlation analysis with optical flow, as shown in the results (see Fig. 11). In each case, the Euclidean distance between different samples was much higher than the distance of an identical sample, compared before and after stretching. All specimen measured before stretching could be identified after stretching (100% detection) for every degree of stretching. But the optical flow approach is limited to small data sets. In case of large data sets, there is an increasing risk for “false positive” results, i.e. two measurements from different components will be incorrectly identified as two measurements from the same component. This can be avoided by application of the SCNN approach. Even in case of the large data set described in the results, a 100% identification of each blank could be achieved in each situation, i.e. for each parameter set. Nevertheless, for formed components the recognition rate was below 90%. This was due to temperature variations, which had an adverse effect on the recognition rate. The temperature-related signal drift has to be compensated in the future. Furthermore, the resolution of the array needs to be improved to achieve better recognition rates.

## Conclusion

Electromagnetic NDE methods are capable of characterizing and identifying an individual production part within a sheet metal processing line. The 3MA method can be used for nondestructive characterization of mechanical properties of sheet metal parts. This can be used not only for quality control at the end of production line, but also at the beginning on cut blanks or even complete coils. With these information it will be possible to predict the behaviour of an individual sheet in subsequent forming process for the purpose of a feed-forward control. In general, it is aimed to determine the forming limit and the spring-back in order to avoid any defects, like cracks, neckings, wrinkles or shape deviations during forming. Based on the 3MA information, it will be possible to exclude “high risk” material from further processing in order to avoid the production of scrap or a machine breakdown resulting in a costly production stoppage. Moreover, the formability information determined with 3MA could be used to adjust the parameters of the forming machine to each individual sheet. This makes it possible to reduce the forming defects described above. Finally, such a feed-forward control of the forming process would allow to use material grades of lower specification, i.e. cheaper material grades in order to reduce costs. In order to use initially determined sheet properties for downstream production steps, e.g. forming but also welding and coating, the individual sheet also have to be traceable.

On the other side, it seems to be possible to track a sheet during several processing steps (cutting, forming, painting) based on a spatially resolved eddy-current (EC) method. This traceability is an essential enabler for closed material cycles. In the area of sheet metal forming, remanufacturing, i.e. the production of new components from old sheet metal components without having to go through the production of a new semi-finished sheet metal product, is currently the preferred approach. This approach could be implemented supported by the EC traceability system. The combination of sensor-based material characterization with a sensor-based intrinsic traceability system could enable completely new concepts for self-controlled, agile production in metal processing.

### Supplementary Information

Supplementary Information.

## Data Availability

The datasets generated during and/or analysed during the current study are available from the corresponding author on reasonable request. See also Supplementary Information.
