# Supplementary material for: Nondestructive material characterization and component identification in sheet metal processing with electromagnetic methods
Source: Sci Rep. 2024 Mar 15;14:6274. doi: 10.1038/s41598-024-55927-4 (PMC10943064; doi:10.1038/s41598-024-55927-4)
Supplement: Supplementary file 1 — Supplementary Information. [file 41598_2024_55927_MOESM1_ESM.docx]

**Supplementary Information**

**Nondestructive material characterization and component identification in sheet metal processing with electromagnetic methods**

**Bernd Wolter^1,*^, Benjamin Straß^1^, Kevin Jacob^1^, Markus Rauhut^2^, Thomas Stephani^2^, Matthias Riemer^3^, and Marko Friedemann^3^**

^1^Fraunhofer Institute for Nondestructive Testing IZFP, 66123 Saarbrücken, Germany

^2^Fraunhofer Institute for Industrial Mathematics ITWM, 67663 Kaiserslautern, Germany

^3^Fraunhofer Institute for Machine Tools and Forming Technology IWU, 09126 Chemnitz, Germany

* [bernd.wolter@izfp.fraunhofer.de](mailto:corresponding.author@email.example)

**Appendix 1: 3MA measuring parameters**

The following tables 1 to 4 show and describe the 3MA measuring parameters.

**Table 1.** Measuring parameters from multi-frequency Eddy Current (EC).

| **Measuring Parameter** | **Identifier** | **Unit** | **Description** |
| --- | --- | --- | --- |
| Re_1_–Re_4_ | $Re1-$Re4 | (V) | Real parts of EC signals at frequencies 1, 2, 3 and 4. |
| Im_1_–Im_4_ | $Im1-$Im4 | (V) | Imaginary parts of EC signals at frequencies 1, 2, 3 and 4. |
| Mag_1_–Mag_4_ | $Mag1-$Mag4 | (V) | Signal magnitudes at frequencies 1, 2, 3 and 4. |
| Ph_1_–Ph_4_ | $Ph1-$Ph4 | (°) | Signal phases at frequencies 1, 2, 3 and 4. |

**Table 2.** Measuring parameters from Harmonic Analysis (HA).

| **Measuring Parameter** | **Identifier** | **Unit** | **Description** |
| --- | --- | --- | --- |
| A_1_ | - | (A/cm) | Amplitude of the fundamental wave (usually not used as a parameter) |
| A_3_, A_5_, A_7_ | $A3, $A5, $A7 | (A/cm) | Amplitudes of the 3rd, 5th and 7th harmonics. |
| P_3_, P_5_, P_7_ | $P3, $P5, $P7 | (°) | Phases of the 3rd, 5th and 7h harmonics. |
| UHS | $UHS | (A/cm) | Sum of all upper harmonics, $UHS=A_{3}+A_{5}+A_{7}+A_{9}$ |
| K | $K | (%) | Distortion factor, $K=100\%*\surd(A_{3}^{2}+A_{5}^{2}+A_{7}^{2})/A_{1}^{2}$ |
| H_CO_ | $Hco | (A/cm) | Coercive magnetic field (coercivity) from harmonic analysis |
| H_RO_ | $Hro | (A/cm) | Harmonic content of the magnetic field strength at zero crossing |
| V_MAG_ | $Vmag | (V) | Final stage voltage of the electromagnet |

**Table 3.** Measuring parameters from Incremental Permeability (IP).

| **Measuring Parameter** | **Identifier** | **Unit** | **Description** |
| --- | --- | --- | --- |
| µ_MAX_ | $umax | (V) | Maximum amplitude |
| µ_MEAN_ | $umean | (V) | Amplitude averaged over one magnetization cycle |
| µ_R_ | $ur | (V) | Amplitude at remanence point |
| H_Cµ_ | $Hcu | (A/cm) | Coercivity from Incremental Permeability (H at µ = µ_Max_) |
| ΔH25_µ_ | $DH25u | (A/cm) | Curve width at 25% of µ_MAX_ |
| ΔH50_µ_ | $DH50u | (A/cm) | Curve width at 50% of µ_MAX_ |
| ΔH75_µ_ | $DH75u | (A/cm) | Curve width at 75% of µ_MAX_ |

**Table 4.** Measuring parameters from Barkhausen Noise (BN).

| **Measuring Parameter** | **Identifier** | **Unit** | **Description** |
| --- | --- | --- | --- |
| M_MAX_ | $Mmax | (V) | Maximum amplitude |
| M_MEAN_ | $Mmean | (V) | Amplitude averaged over one magnetization cycle |
| M_R_ | $Mr | (V) | Amplitude at remanence point |
| H_CM_ | $Hcm | (A/cm) | Coercivity from Barkhausen Nois (H at M = M_Max_) |
| ΔH25_M_ | $DH2m | (A/cm) | Curve width at 25% of M_MAX_ |
| ΔH50_M_ | $DH50m | (A/cm) | Curve width at 50% of M_MAX_ |
| ΔH75_M_ | $DH75m | (A/cm) | Curve width at 75% of M_MAX_ |

**Appendix 2: Calibration functions**

The following tables 5 and 6 shows the calibration functions and the calibration results used for sheet material characterization with 3MA. For the description of the identifiers see tables 1 to 4. The calibration results show the correlations between the material parameters determined with the non-destructive 3MA method and the material parameters determined with destructive laboratory methods.

**Table 5.** Calibration functions for sheet material characterization with 3MA.

| **Hardness, H (HV)** | **Yield strength, Rp0.2 (MPa)** | **Tensile strength, Rm (MPa)** |
| --- | --- | --- |
| -7.9911652781E+1*(1)  -1.6367525628E+1*($P3)  +2.7875884282E+3*($umax)  -6.3790001234E+3*($umean)  +3.8663477034E+0*($Hcu)  +1.2028457586E+2*(sqrt(abs(($A3))))  +5.0905481681E+0*(sqrt(abs(($P7))))  +1.6080891127E+2*(sqrt(abs(($ur))))  -2.1744303080E+0*(sqrt(abs(($Hcu))))  +1.3555568001E+1*(sqrt(abs(($DH50u)))) | 2.3108658420E+2*(1)  +3.1298398587E+2*($P3)  +1.5565130125E+1*($P7)  +9.8445158053E+3*($umean)  +1.6246058972E+1*($Hcu)  -3.8380388815E+2*(sqrt(abs(($A5))))  -2.2323206317E+2*(sqrt(abs(($A7))))  -7.5680089988E+2*(sqrt(abs(($P3))))  +6.7822829691E+2*(sqrt(abs(($UHS))))  -8.8897482734E+0*(sqrt(abs(($Hcu)))) | 2.2655679943E+2*(1)  +4.7428065436E+2*($A3)  -2.4461837708E+1*($P7)  +8.7122788809E+0*($Hco)  +5.6760273319E+3*($umax)  +8.6551102513E+1*(sqrt(abs(($P7))))  -1.1172448886E+2*(sqrt(abs(($K))))  -1.9089459956E+3*(sqrt(abs(($umean))))  +1.0643736525E+1*(sqrt(abs(($Hcu))))  +1.9682473789E+1*(sqrt(abs(($DH25u)))) |
| **Elongation, A (%)** | **r-value (-)** | **n-value (-)** |
| 1.6052897116E+2*(1)  -9.5048079935E+1*($A3)  +2.8109414933E+1*($UHS)  -4.4312969502E+1*($Mmean)  -1.8689287121E+2*($umax)  +1.9258580392E+3*($ur)  -8.8740404107E-1*($Hcu)  -4.9347029772E+0*(sqrt(abs(($P7))))  -7.8874137368E+2*(sqrt(abs(($ur))))  -3.4120866898E+0*(sqrt(abs(($DH75u)))) | 6.9434081391E+0*(1)  -1.0705901714E+1*($A3)  +2.4260422956E+1*($A5)  +7.2802297757E-1*($P7)  -1.0787840895E-1*($Hcu)  -1.3312232217E+1*(sqrt(abs(($A5))))  -2.3972844451E+0*(sqrt(abs(($P7))))  +2.6953052275E+0*(sqrt(abs(($UHS))))  +1.0574635334E+0*(sqrt(abs(($K))))  -1.0509949962E+1*(sqrt(abs(($ur)))) | 5.5184158361E-1*(1)  -8.8100627919E-1*($A3)  +1.5980885602E-2*($P5)  -6.0760624323E-2*($Hro)  +5.3268872448E-4*($DH75m)  -2.7746521100E+0*($ur)  -3.4681924231E-3*($Hcu)  -1.6150072667E-3*($DH50u)  -9.6084785790E-3*(sqrt(abs(($P7))))  -4.4321754691E-3*(sqrt(abs(($DH25m)))) |

**Table 6.** Calibration results for sheet material characterization with 3MA.

| **Hardness, H (HV)** | **Yield strength, Rp0.2 (MPa)** | **Tensile strength, Rm (MPa)** |
| --- | --- | --- |
| 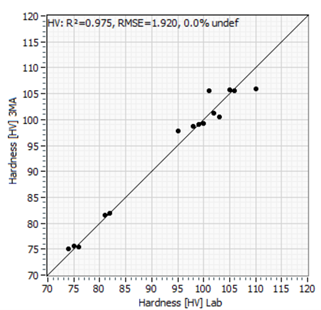 | 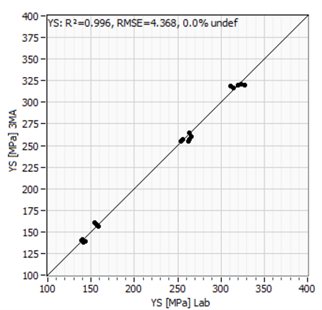 | 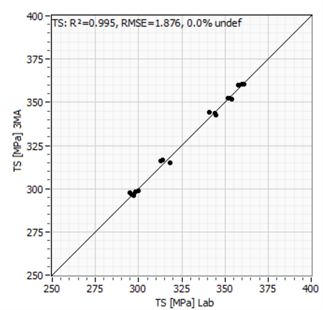 |
| **Elongation, A (%)** | **r-value (-)** | **n-value (-)** |
| 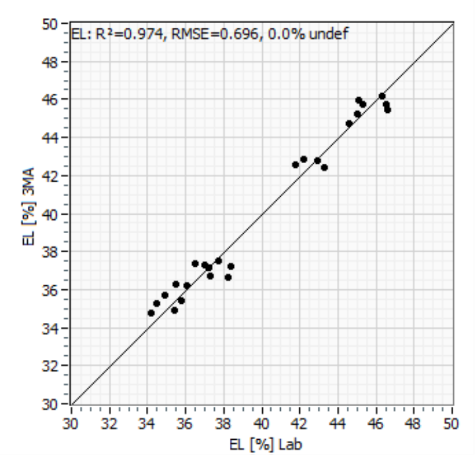 | 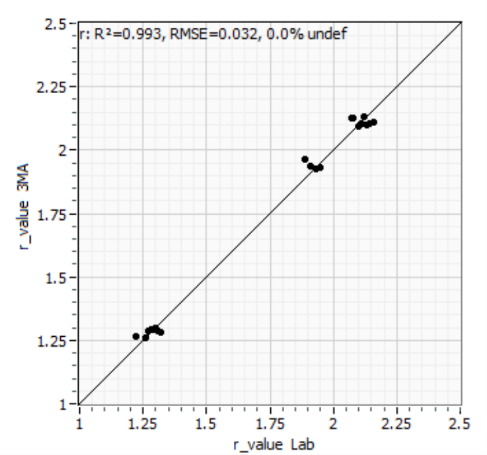 | 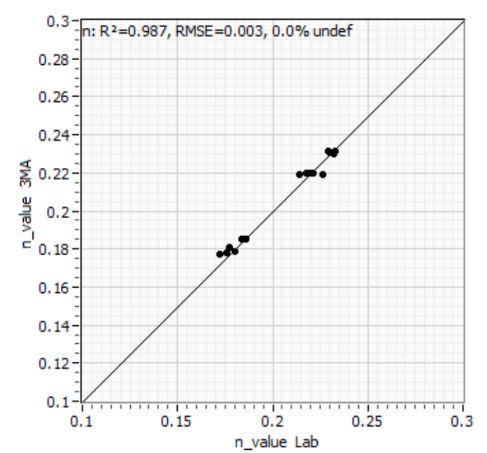 |

**Appendix 3: Results from sheet metal part identification of all 3MA measuring parameters**

The following table 7 shows the results of initial measurements with the 3MA system on a flat sample before and after plastic deformation (stretching). Here, all 3MA parameters are shown. The highest recognition rate has been achieved with the parameters Re_3_, Mag_3_ and Mag_4_ from EC method.

**Table 7.** Results of all 3MA measuring parameters before and after stretching.

| **3MA**  **parameter** | **Result of a measurement before**  **stretching** | **Result of a 1^st^ measurement after**  **stretching (no shifting)** | **Result of a 2^nd^ measurement after**  **stretching (slightly shifted sample)** |
| --- | --- | --- | --- |
| Re_1_ | 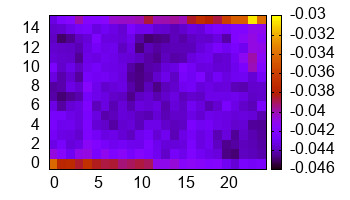 | 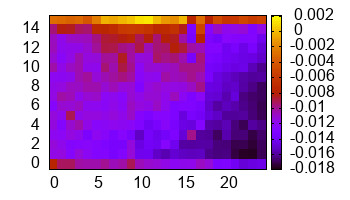 | 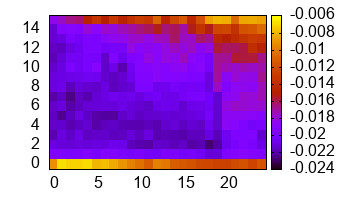 |
| Im_1_ | 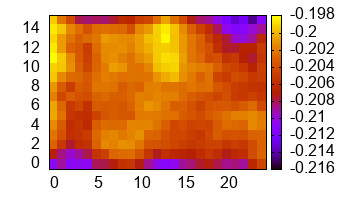 | 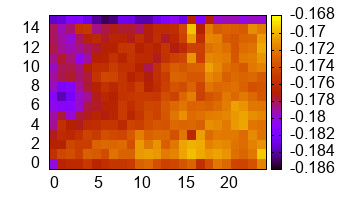 | 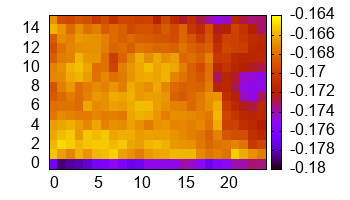 |
| Mag_1_ | 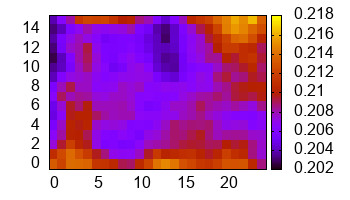 | 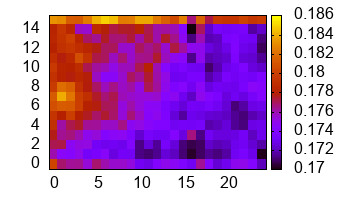 | 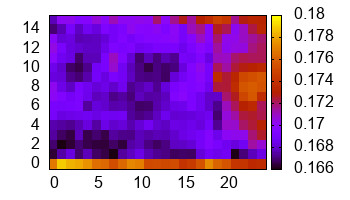 |
| Ph_1_ | 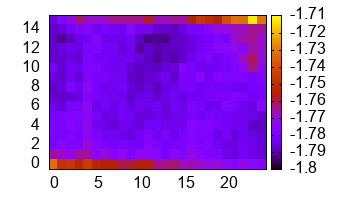 | 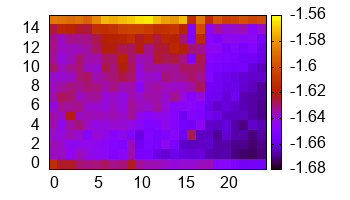 | 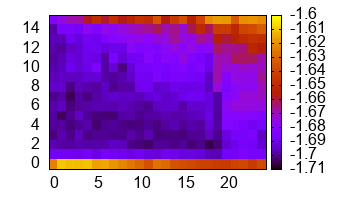 |
| Re_2_ | 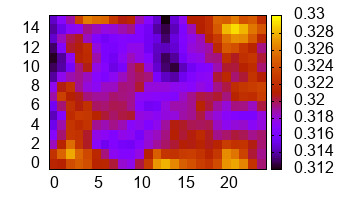 | 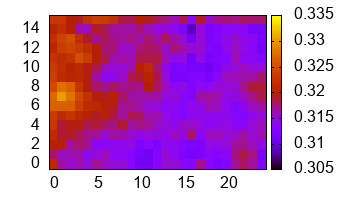 | 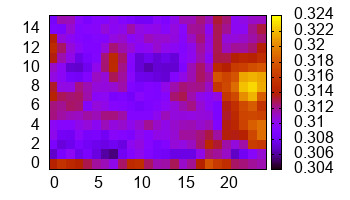 |
| Im_2_ | 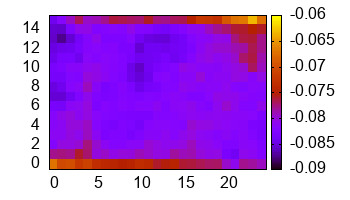 | 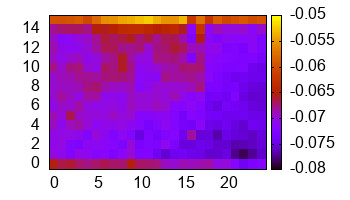 | 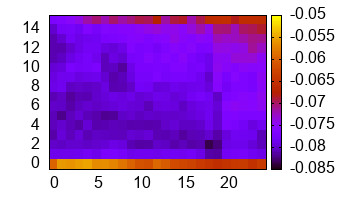 |
| Mag_2_ | 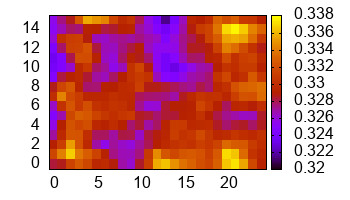 | 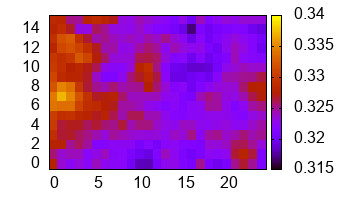 | 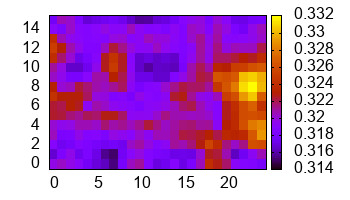 |
| Ph_2_ | 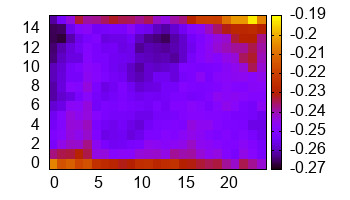 | 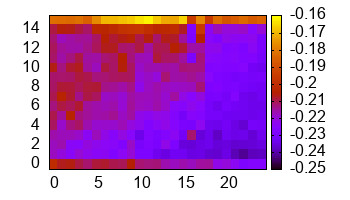 | 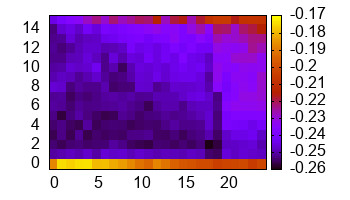 |
| Re_3_ | 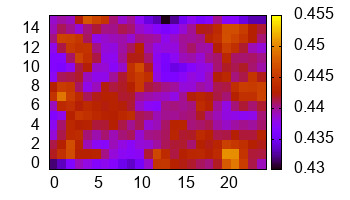 | 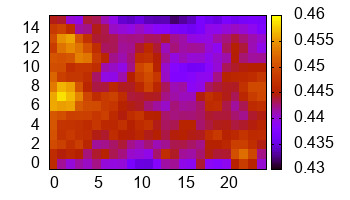 | 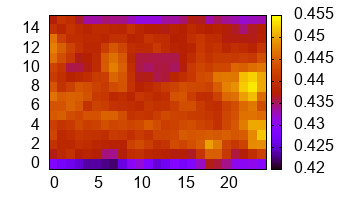 |
| Im_3_ | 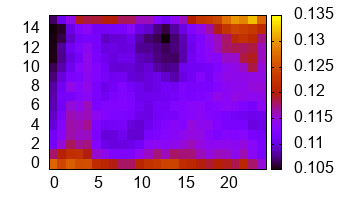 | 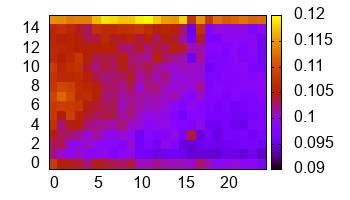 | 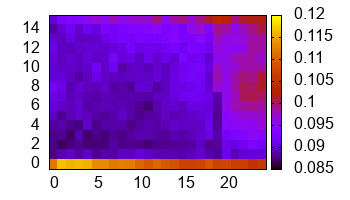 |
| Mag_3_ | 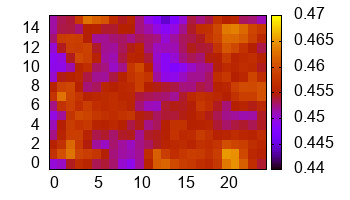 | 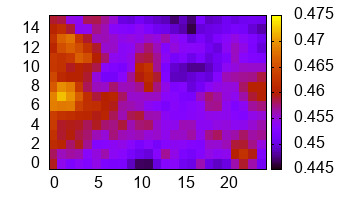 | 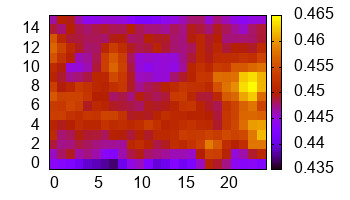 |
| Ph_3_ | 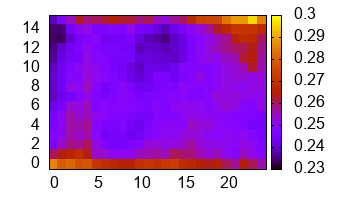 | 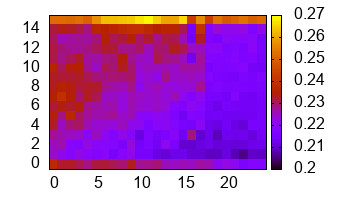 | 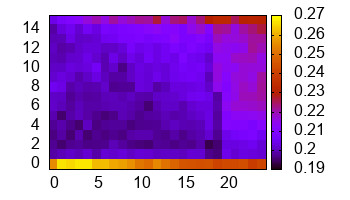 |
| Re_4_ | 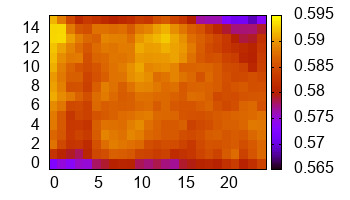 | 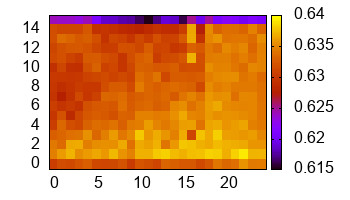 | 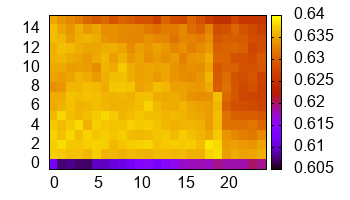 |
| Im_4_ | 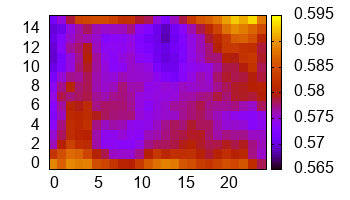 | 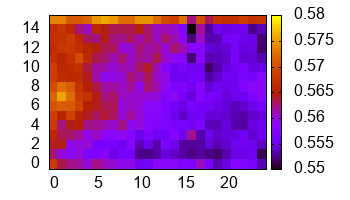 | 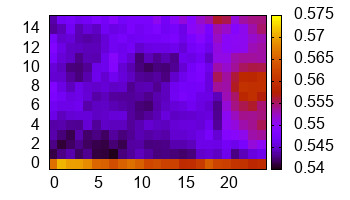 |
| Mag_4_ | 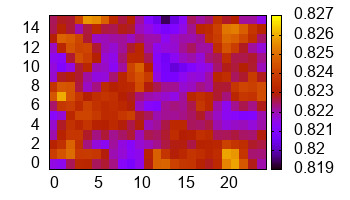 | 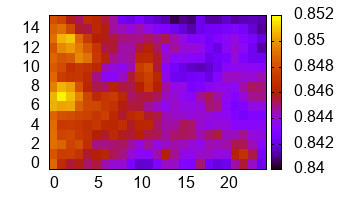 | 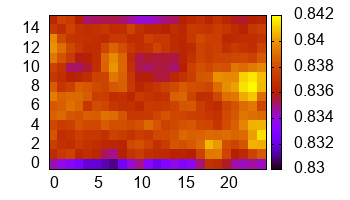 |
| Ph_4_ | 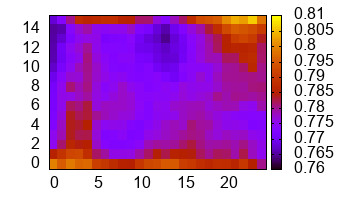 | 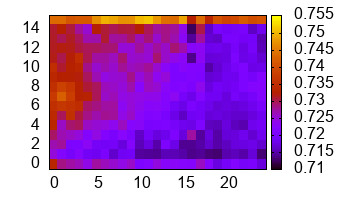 | 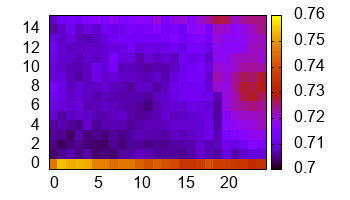 |
| V_MAG_ | 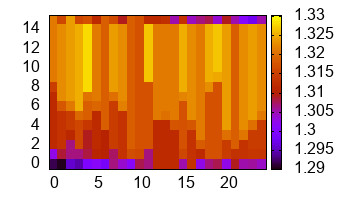 | 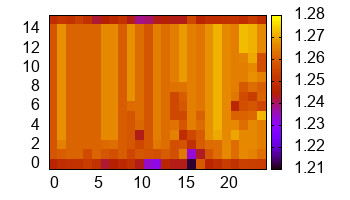 | 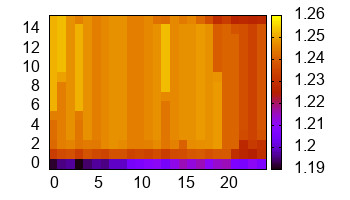 |
| A_3_ | 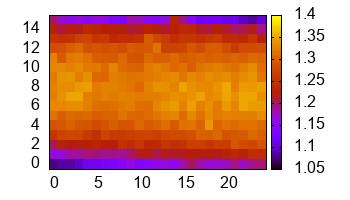 | 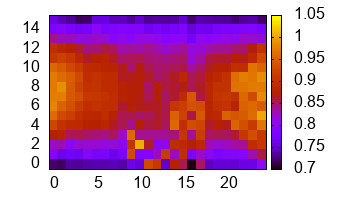 | 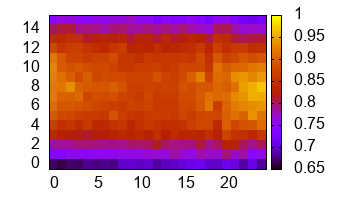 |
| A_5_ | 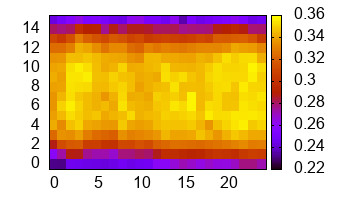 | 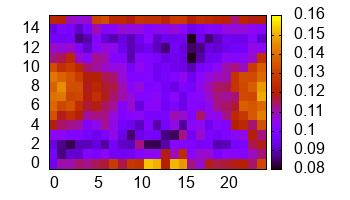 | 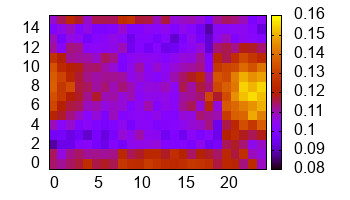 |
| A_7_ | 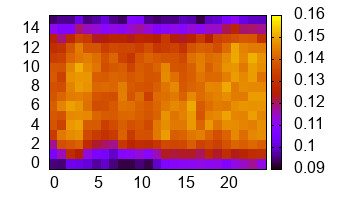 | 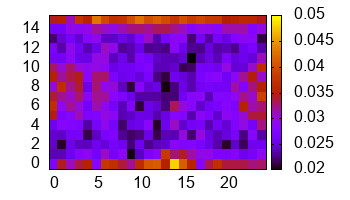 | 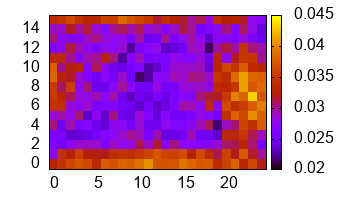 |
| P_3_ | 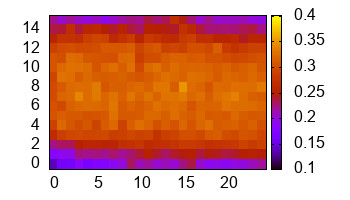 | 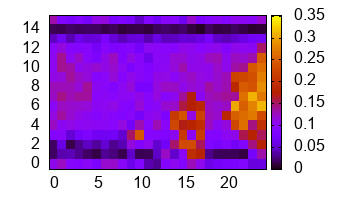 | 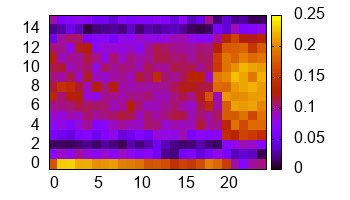 |
| P_5_ | 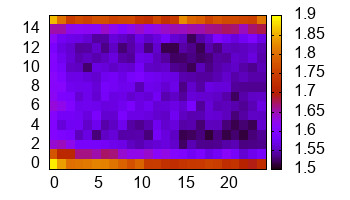 | 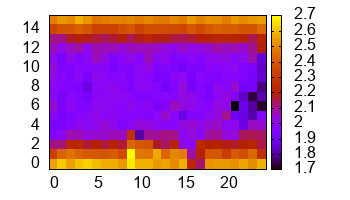 | 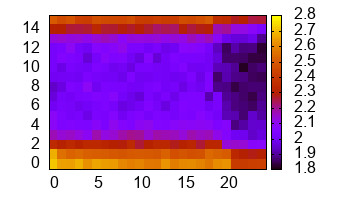 |
| P_7_ | 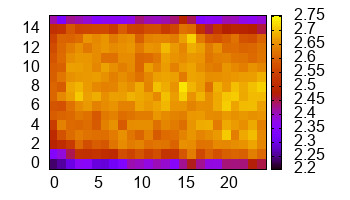 | 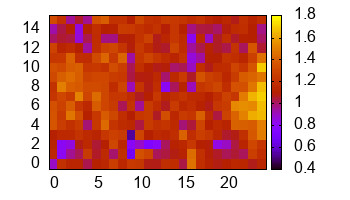 | 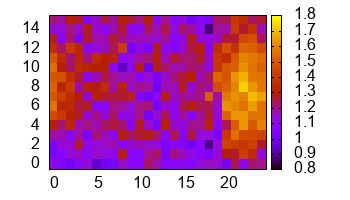 |
| UHS | 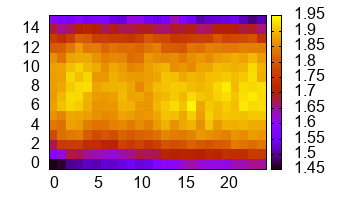 | 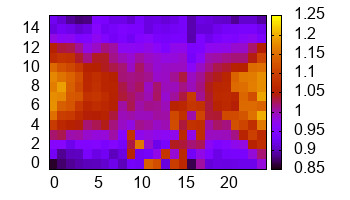 | 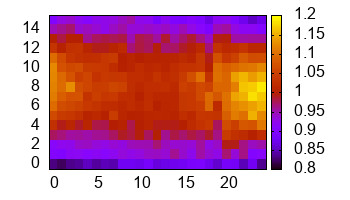 |
| K | 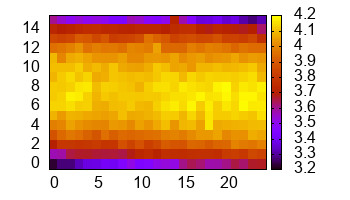 | 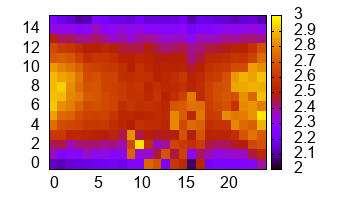 | 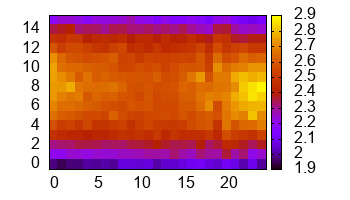 |
| H_CO_ | 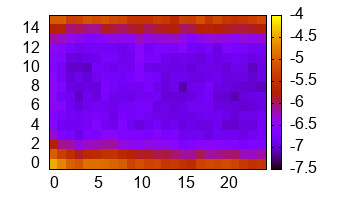 | 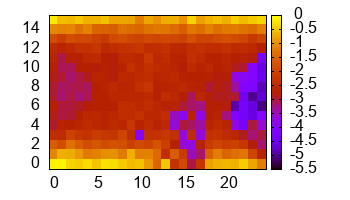 | 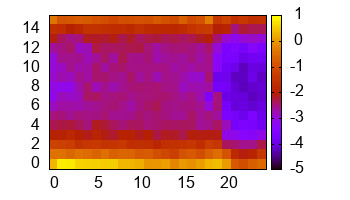 |
| H_RO_ | 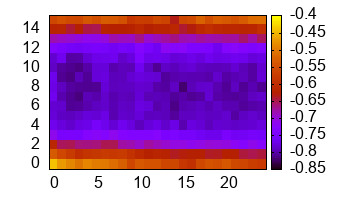 | 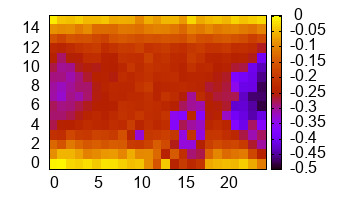 | 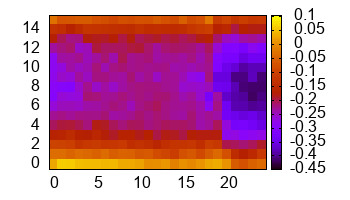 |
| µ_MAX_ | 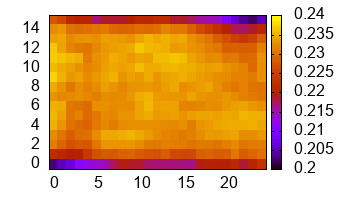 | 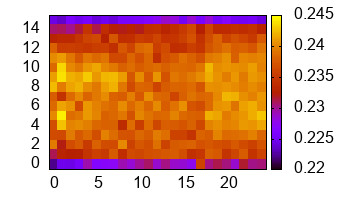 | 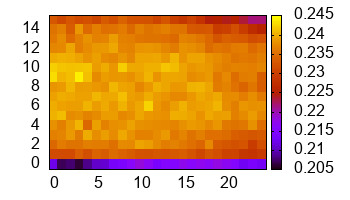 |
| µ_MEAN_ | 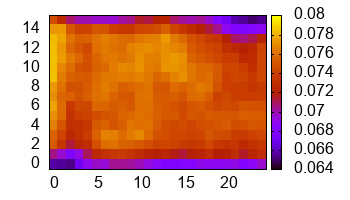 | 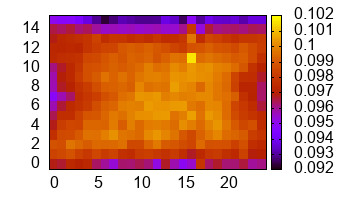 | 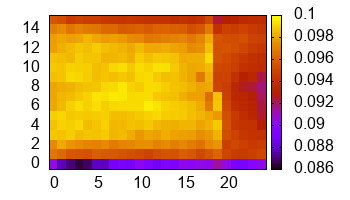 |
| µ_R_ | 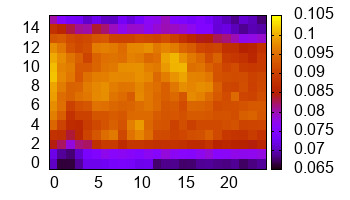 | 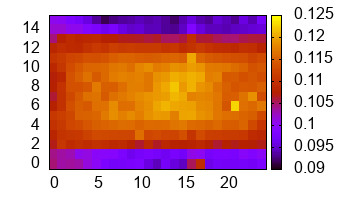 | 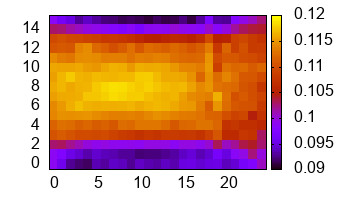 |
| H_Cµ_ | 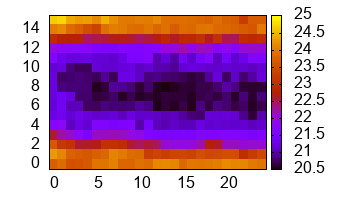 | 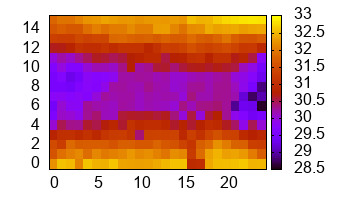 | 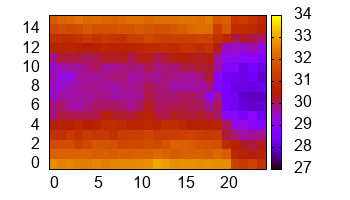 |
| DH25_µ_ | 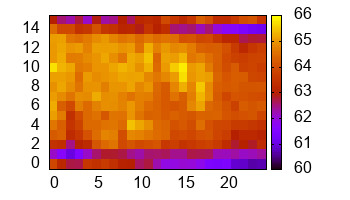 |  |  |
| DH50_µ_ |  |  |  |
| DH75_µ_ |  |  |  |
| M_MAX_ |  |  |  |
| M_MEAN_ |  |  |  |
| M_R_ |  |  |  |
| H_CM_ |  |  |  |
| DH25_M_ |  |  |  |
| DH50_M_ |  |  |  |
| DH75_M_ |  |  |  |
